# Classification and statistical analysis of structural disorder in crystalline materials

**DOI:** 10.1107/S1600576725003000

**Published:** 2025-05-29

**Authors:** Dmytro Antypov, Chris M. Collins, Matthew S. Dyer, John B. Claridge, Matthew J. Rosseinsky

**Affiliations:** a Leverhulme Research Centre for Functional Materials Design Materials Innovation Factory University of Liverpool Liverpool UK; b Department of Chemistry University of Liverpool Liverpool UK; University of Silesia in Katowice, Poland

**Keywords:** disorder classification, CIFs, entropy calculation, mixing entropy, configurational entropy

## Abstract

Approximately 50% of entries in the Inorganic Crystal Structure Database (ICSD; https://www.fiz-karlsruhe.de/en) exhibit some form of structural disorder. This work aims to provide a thorough analysis of structurally disordered materials within the ICSD, using data extracted from crystallographic information files. To achieve this, we derive a classification of structurally disordered crystalline materials described by their spatially averaged structures and introduce a range of quantitative measures of structural disorder. The overarching aim of this classification and analysis is to facilitate high-throughput and machine learning studies of disordered materials. To demonstrate the application of our approach, we perform statistical analysis of the disordered compounds reported in the ICSD to identify general trends in the distribution of disorder across different chemical elements, structures and classes of materials.

## Abstract 2

We have developed a new classification system for disorder in crystalline materials based on analysis of the information present in crystallographic information files. By recognizing the role of the proximity of crystallographic sites to each other and the interplay of partial occupancies in creating disorder, we consider seven distinct types of disordered orbits and propose modifications to the way the entropy of disordered systems is calculated.

## 1. Introduction

Disorder, defined as a deviation from order (Ziman, 1979 ▸), can manifest in various forms and degrees. In crystalline materials, disorder in the types and positions of atoms, in the orientations of molecules or multi-atomic sub-units, or in atomic magnetic spins or dipolar moments may coexist with long-range structural order. Disordered materials are fairly common, with approximately 50% of entries in the Inorganic Crystal Structure Database (ICSD; https://www.fiz-karlsruhe.de/en) (Zagorac *et al.*, 2019 ▸) containing disorder as a structural feature. We specifically focus here on disorder in inorganic materials; other disordered systems such as amorphous polymers, glasses and organic crystals are not considered in this work.

Disorder affects the properties of materials. Classical examples include a decrease in thermal conductivity upon introduction of structural disorder (Ghosh *et al.*, 2022 ▸), or higher ionic conductivity in compounds with Li-ion site disorder compared with ordered compounds (Jonderian & McCalla, 2021 ▸). The utilization of disorder as an instrument in the design of functional materials advances in parallel with a deeper understanding of structure–property relationships in disordered materials (Simonov & Goodwin, 2020 ▸). For example, in spinel ferrites, (Zn_1−*x*_Fe_*x*_)[Zn_*x*_Fe_2−*x*_]O_4_, site disorder can be used to control photocatalytic activity (Li *et al.*, 2019 ▸). Substitutional disorder is used to enhance ionic conductivity in Li-ion solid conductors, by means of introducing Li-ion vacancies upon aliovalent doping and lowering the activation energy (Feng *et al.*, 2020 ▸), and to improve cathode materials, for example to design a Co-free cathode material LiNi_0.5_Mn_0.43_Ti_0.02_Mg_0.02_Nb_0.01_Mo_0.02_O_2_ with high cycling stability due to a complex concentrated doping strategy (Zhang *et al.*, 2023 ▸). More generally, disorder can also be used as a stabilization mechanism to access novel materials, which was demonstrated through the discovery of high-entropy alloys and ceramics (Miracle & Senkov, 2017 ▸; George *et al.*, 2019 ▸; Oses *et al.*, 2020 ▸).

Systematic large-scale high-throughput studies aiming to discover new disordered materials are still rare. One of the reasons is that, although approaches to simulating disordered materials to predict their thermodynamic stability and properties have been developed (Madrid & Ghuman, 2021 ▸; Puchala *et al.*, 2023 ▸; Chang *et al.*, 2019 ▸; Yang *et al.*, 2022 ▸; Grau-Crespo *et al.*, 2007 ▸; Yang *et al.*, 2016 ▸), they remain much more computationally expensive than analogous methods for ordered materials. Machine learning as a complementary approach is a promising way of exploring the complex relationships between disorder in materials and properties connected to it. To help developments in this area, high-quality data sets describing disorder and connecting it to properties are required.

In this paper, we explore one of the most abundant sources of experimental information about disorder in crystalline materials, the ICSD (Zagorac *et al.*, 2019 ▸). The ICSD contains information about the average structures of crystalline mater­ials obtained by X-ray and neutron diffraction. Using this source of information imposes limitations on the types of disorder available for analysis. The possible types of disorder reduce to structural features which are reflected in a crystallographic information file (CIF) (Hall *et al.*, 1991 ▸; Brown & McMahon, 2002 ▸). The first limitation arises because X-ray and neutron diffraction experiments deliver globally averaged information, so correlations and local ordering phenomena cannot be observed. The second limitation is that the positions of hydrogen and other light atoms such as lithium are often omitted (as most of the data were obtained by X-ray diffraction).

In a CIF, a structure is represented as a list of crystallographic orbits, each of which is characterized by the element occupying the orbit and by its oxidation state, multiplicity, Wyckoff symbol, fractional coordinates, site occupancy and anisotropic displacement parameters. Disorder is revealed by fractional site occupancies. At the most general level, if there are any fractional occupancies, the compound is disordered, and it is ordered otherwise.

By combining information on site occupancies with information on the proximity of the sites to each other, we obtain a more detailed description of disorder. Specifically, we assign the positional disorder label (P) to orbits containing sites that intersect because they are too close to each other to be occupied simultaneously. If an individual site or a combined site produced by positional disorder is not fully occupied, we assign it as containing vacancies (V), thus reserving the term ‘positional disorder’ exclusively for those sites that are intersecting. This is a narrower definition than that often used for positional disorder and is made in order to recognize the role of site intersection in creating disorder of this type rather than vacancy disorder. We define ‘vacancy disorder’ as the presence of a combined site with a total occupancy of less than 1, which covers any vacancy unexplained by positional disorder, for example partially occupied interstitials. As a result, we distinguish the following types of structural disorder: substitutional (different elements occupying the same site), positional (the presence of intersecting sites), vacancy formation (total site occupancy is below 1) and their combinations. We have developed a tool that, for a given CIF, assigns labels to each crystallographic orbit to classify the type of disorder it contains. This classification of orbits allows us to classify complete crystal structures according to the type of disorder they harbour and to study the distribution of disorder in the ICSD.

Introducing a disorder classification in inorganic materials based on the disorder classification of orbits aligns well with the general guidance on determining structure types in in­organic materials (Lima-De-Faria *et al.*, 1990 ▸; Allmann & Hinek, 2007 ▸; Hicks *et al.*, 2021*b* ▸) and may help to distinguish different disordered structure types in materials where disorder is common and plays an important role, such as spinels, argyrodites and other crystalline ion conductors.

To quantify the disorder we propose to use the fraction of disordered sites (either for a particular disorder type or for all disorder types combined) in the compound and review the expressions for mixing and configurational entropies (De Souza & Harrington, 2023 ▸). These quantitative measures allow comparison of the degree of disorder in materials. As our calculations are based on crystallographic average structures, the resulting entropies represent the upper bound of the true values of the configurational or mixing entropies, which will be reduced by any local correlations present.

Facilitated by the disorder classification, we analyse the distribution of different types of disorder in the ICSD. The consequent understanding of the general trends in the different types of disorder with respect to elements and structures provides useful insights for future studies and the design of disordered materials.

**Figure 1:**
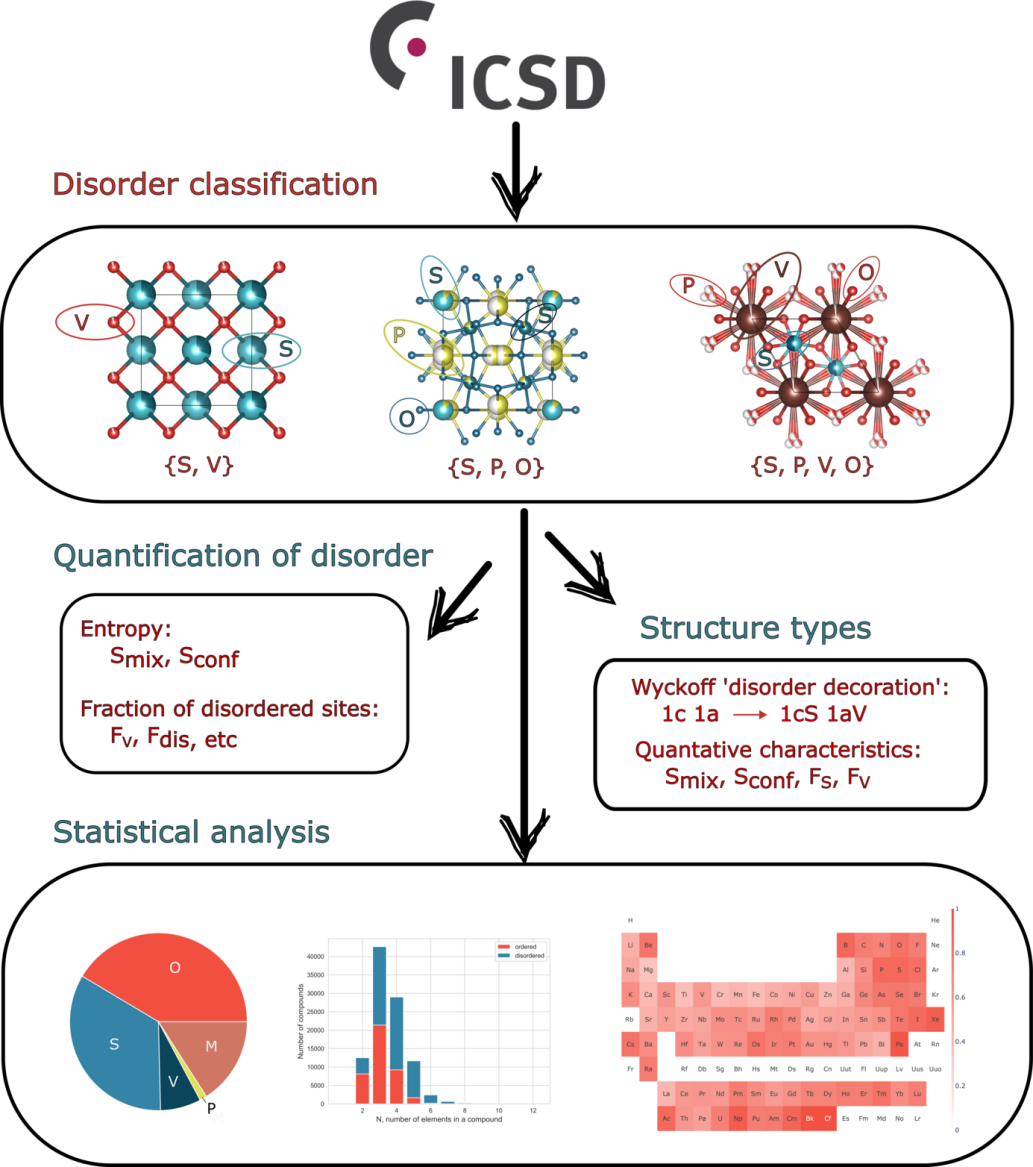
In this paper we analyse the statistical distribution of disorder in the ICSD. To do this, we (i) introduce a method of classification of crystallographic orbits with respect to the type of disorder they contain, using labels O (order), S (substitutional), V (vacancies) and P (positional), (ii) introduce quantitative measures of disorder such as mixing and configurational entropy, fraction of vacancies, and fraction of disordered sites, and (iii) propose how the description of disorder can be added to the description of structure types, as 50% of compounds in the ICSD contain disorder.

The paper is organized as follows (see Fig. 1 ▸). First, we discuss the quantitative and qualitative characteristics of disorder. To do so, we introduce different disorder types for the classification of orbits based on the information available in a CIF. We discuss how compounds can be classified with respect to the disorder they contain and how the description of structure types can be modified to include information about disorder. Then we study the distribution of the introduced disorder types in the ICSD. We are mostly interested in the distribution over elements, because they determine interactions, and over structures, defining underlying geometries, as any model of disorder contains these two components (Simonov & Goodwin, 2020 ▸). We analyse the statistics of these distributions in different classes of materials. Finally, we analyse the statistics of the distribution of elements on substitutionally disordered orbits and juxtapose it with the Pettifor scale (Pettifor, 1984 ▸; Glawe *et al.*, 2016 ▸), which describes the ability of elements to substitute each other in ordered compounds without causing a change in structure type. We end with conclusions.

## 2. Qualitative and quantitative characteristics of disorder

At the highest level of classification, all the richness of experimental compounds in the ICSD can be reduced to two classes, ordered and disordered, based on the presence of partial occupancies in a CIF. If a compound contains occupancies smaller than 1 for any element listed it is disordered. Otherwise it is ordered. Here we consider only entries at atmospheric pressure and room temperature (270 < *T* < 310 K) which have identical lists of elements in the composition formula and the structural part of the CIF and which do not contain hydrogen (which amounts to ∼64% of all entries in the ICSD). More details about data preparation can be found in Section 2.1; retaining compounds with hydrogen would give ∼70% of all entries. Later in the paper we refer to this data set as ‘the ICSD’. When analysing the data in Section 2.4, we additionally remove duplicates, which we define as compounds with the same composition and the same space group type number. Only the duplicate with the largest configurational entropy is retained in the data set (see Section 2.3 for the definition of configurational entropy).

### 2.1. Data preparation

Experimental CIFs were downloaded from the ICSD in March 2024 with the help of *ICSDClient* (https://github.com/lrcfmd/ICSDClient), returning 221688 entries. Approximately 10% of CIFs failed to be processed for reasons such as non-standard formatting, unphysical occupancies [all CIFs with occupancies for orbits larger than 1.05 were discarded, while those in the range (1, 1.05) were re-scaled to 1], the presence of element symbols which are not in the periodic table (generic letters like X or M) or entries with a reported composition different from the composition found in the structure. After excluding entries with hydrogen (there were approximately 9% of such entries) and entries with more than 500 crystallographic sites (there were approximately 0.4% of such entries), we arrived at 176792 entries. We kept only compounds measured under ambient conditions, which we define as *T* = 270–310 K and *P* < 0.11 MPa. This reduced the number of entries to 140903.

For the data analysis conducted in Section 2.4, duplicates with the same composition and space group were removed. Unless stated otherwise, we retained a single entry in each case, selected according to the largest entropy for a given composition and space group. This duplicate-free data set contained 101662 entries.

To prepare the oxide data set we filtered out all compounds containing hydrogen and any anions but oxygen. An analogous method was used to create the data sets for carbides, nitrides, fluorides, phosphates, sulfides, chlorides, selenides, bromides and iodides.

To create the intermetallics/alloys data set, we retained compounds containing only metals in their composition formula.

### 2.2. Classification of crystallographic orbits

By using the information stored in the CIF about the occupancy and location of atomic sites, we distinguish different types of structural disorder. Our classification of structural disorder is based on assigning different disorder labels to the crystallographic orbits described in a CIF.

A crystallographic orbit is a set of points which are generated from one site by the symmetry operations of the space group *G* (Souvignier, 2016 ▸). The space group *G* is called the generating space group of the orbit. The orbit is completely determined by its points in the unit cell. In a CIF, each orbit is specified by the fractional coordinates, multiplicity, Wyckoff symbol, species occupying it, value of occupancy by species and atomic displacement parameters.

With respect to disorder type, we distinguish ordered orbits (O), orbits with substitutional disorder (S), orbits with positional disorder (P), orbits with vacancies (V) and mixed types: a combination of substitutional disorder and vacancies (SV), a combination of substitutional disorder and positional disorder (SP), a combination of positional disorder and vacancies (VP), and a combination of positional and substitutional disorder and vacancies (SVP).

Table 1 ▸ shows the way the classification works. To decide which type of disorder to assign to the orbit, we consider six parameters: (i) the number of different elements occupying the orbit, or orbits in the case of their intersection, (ii) the total occupancy, which is defined for each crystallographic site as the sum of occupancies over all the elements located at this site, (iii) whether there is internal intersection (defined below) of sites within the orbit, (iv) whether there is external intersection of sites of a given orbit with the sites of another orbit, (v) ‘the total occupancy of the combined site’ which is the sum of the occupancies of crystallographic sites that intersect, and (vi) whether the elements occupying the intersecting orbits are the same or not.

We say that two sites intersect if the distance between them 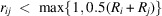, where the distance is measured in ångströms, and *R*_*i*_ and *R*_*j*_ are, respectively, the average ionic radii of species occupying crystallographic sites *i* and *j*. The ionic radii are taken from Shannon’s tables of ionic radii (Shannon, 1976 ▸). The oxidation states of the species are taken from the CIF. Since the coordination number is not determined in a CIF, the smallest radius is taken for a given oxidation state of an element. If the ionic radius of a species is not available in Shannon’s table (for example, if the oxidation state is zero), the atomic radius (Slater, 1964 ▸) is used instead. More details and explanations of the choice of criteria for intersection of sites are given in the supporting information. We combine all of the neighbouring intersecting sites into a combined site. Thus, a combined site is a group of intersecting sites that do not intersect with other combined sites. More formally, if we represent sites as nodes and intersections as edges of a graph, then each connected component of this graph is defined as a ‘combined site’.

We define internal intersection for a given crystallographic orbit as a situation when at least two sites belonging to this orbit intersect. In order to deal with internal intersections, we combine intersecting sites into combined sites, which leads to a corresponding decrease in the combined orbit multiplicity, as the total number of atoms on the orbit remains unchanged. For each orbit with internal intersection we define what the combined sites are and calculate their occupancy and multiplicity.

We say that two different orbits intersect externally if any sites belonging to these two orbits intersect. In order to deal with the external intersection of two or more orbits, we combine the intersecting orbits into a single combined orbit. We use ‘combined orbit’ and ‘orbit’ interchangeably, in contrast to the traditional meaning of the crystallographic orbit given above. Similar to an internally intersecting orbit, the description of a combined orbit requires the calculation of the total occupancy of the combined site and its multiplicity.

**Figure 2:**
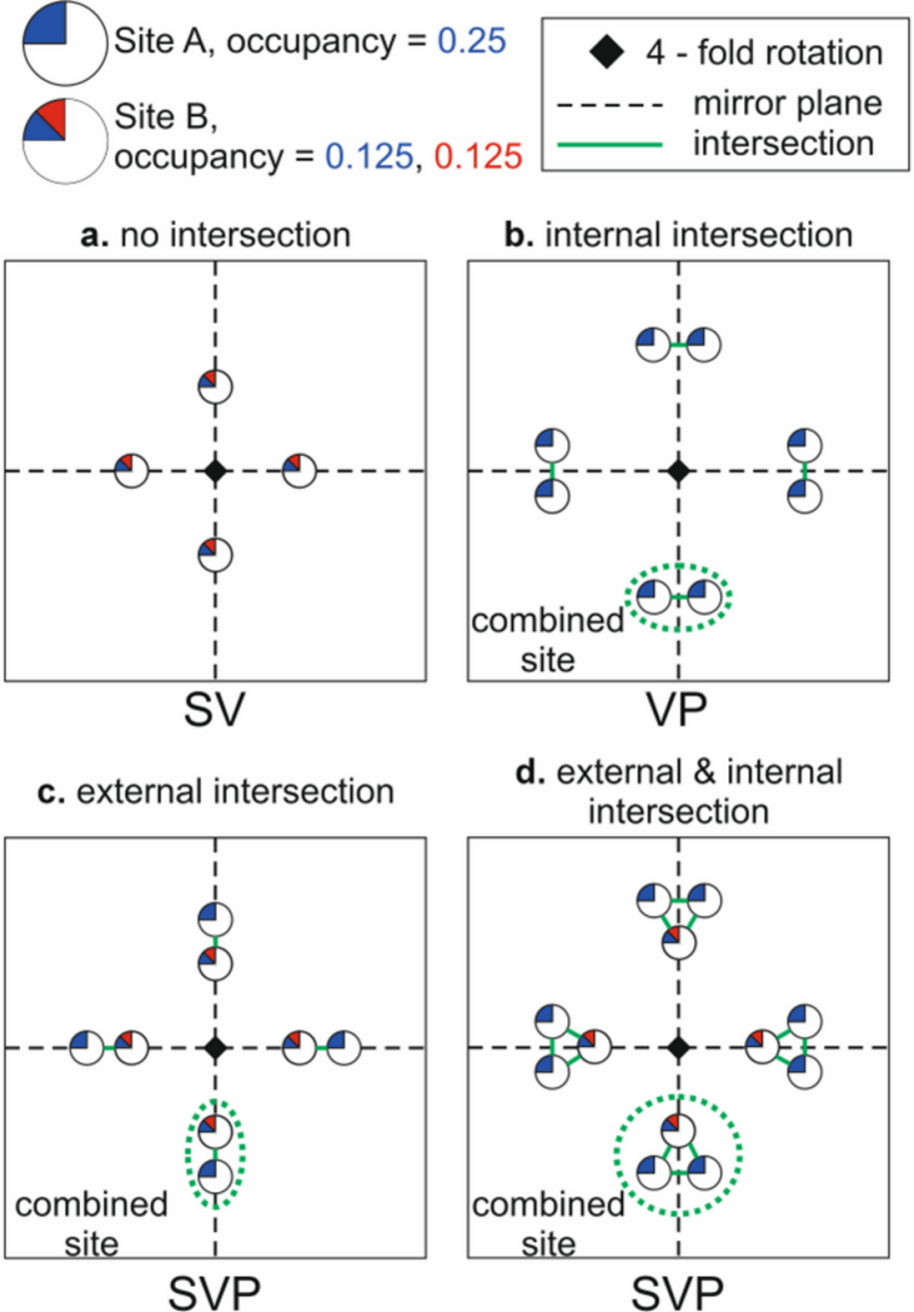
Internal and external intersections. (*a*) An orbit with no intersection, (*b*) an orbit with internal intersection, (*c*) a combined orbit produced by two crystallographic orbits that have no internal intersections, and (*d*) a combined orbit containing both external and internal intersections. When intersecting positions within (*b*) a single orbit or (*c*) and (*d*) multiple orbits form a combined site, then in order to preserve the total number of atoms in the cell while describing the structure in terms of combined sites we introduce the appropriate multiplicity for that site.

To demonstrate examples of internal and external intersections, in Fig. 2 ▸ we consider a 2D unit cell containing two mirror planes and one four-fold rotation axis. We consider two types of crystallographic sites which can be present in our cell: Site *A* which is occupied by the ‘blue’ element and has an occupancy of 0.25, and Site *B* which is occupied by ‘blue’ and ‘red’ elements and has a mixed occupancy of 0.125 blue and 0.125 red. In Fig. 2 ▸(*a*), Site *B* is placed on one of the mirror planes and the symmetry operators generate three additional positions (giving a total multiplicity of 4), which make up the orbit. Since none of these four positions are close to each other, the disorder present comes purely from Site *B*, where we have a site which is not fully occupied and contains two elements, so we define this as substitutional and vacancy disorder (SV). In Fig. 2 ▸(*b*), Site *A* is placed on a general position near to one of the mirror planes and the symmetry operators generate seven additional positions (giving a multiplicity of 8). These eight positions group into four pairs which do not intersect with each other. Since all the intersecting sites are generated by the same crystallographic orbit, we refer to this as internal intersection. We consider each of the pairs of intersecting sites as one combined site, because the distance marked in green is too short for simultaneous occupation of both connected positions, with a total occupancy of 2 × 0.25 = 0.5 blue and a multiplicity of 4. The combined site not being fully occupied results in the classification of positional and vacancy disorder (PV). In Fig. 2 ▸(*c*), both Site *A* and Site *B* have been included, with both sites placed on one of the mirror planes and close enough to each other so they intersect. For both sites, three additional positions are generated when applying the symmetry operators to complete the orbits. Since Site *A* and Site *B* intersect and are from different orbits, we refer to this as an external intersection. Each of the four pairs of intersecting Site *A* and Site *B* creates a combined site with a multiplicity of 4 and an occupancy of 0.375 blue and 0.125 red, and thus contains substitutional disorder. The combined site additionally contains vacancies, as its total occupancy is less than 1 and as it also contains intersecting sites (indicating positional disorder). The combined orbit is thus classified with the symbol SVP. In Fig. 2 ▸(*d*), Site *A* is placed on the same eight-fold site shown in Fig. 2 ▸(*b*) and Site *B* is placed on the same site as shown in Fig. 2 ▸(*a*), so the resulting combined sites now contain both external and internal intersections. The total occupancy of the combined site is now 0.625 blue and 0.125 red. This combined site is another example of an SVP site, resulting from the same reasoning as in Fig. 2 ▸(*c*).

The rules for classification of orbit disorder are shown in Table 1 ▸. Lines 1–4 describe orbits without positional disorder, or in other words, orbits without internal or external intersections. If the orbit is occupied by one element with occupancy 1, then the orbit is ordered (O). If the orbit is occupied by one element but the occupancy is smaller than 1, then this orbit is classified as a vacancy (V) orbit. If the orbit is occupied by more than one element but the total occupancy is close to 1 (we allow a tolerance of 0.011 to account for a possible rounding error), then the orbit is classified as an orbit with substitutional disorder (S). If the orbit is occupied by more than one element and the total occupancy is smaller than 1 minus the tolerance factor, then the orbit is classified as an orbit with substitutional disorder and vacancies (SV).

If internal or external intersection of orbits is present, then we consider the orbit as having positional disorder. First, we consider the case when there is an internal but no external intersection, which is described by lines 5–8 in Table 1 ▸. If there is only one type of element occupying the orbit and the occupancy of the combined site is close to 1 (here and thereafter we again allow for a tolerance of 0.011 to account for a possible rounding error), then the orbit is classified as a purely positionally disordered orbit (P). If there is only one type of element occupying the orbit but the occupancy of the combined site is less than 1 minus the tolerance factor, then the orbit is classified as having both positional disorder and vacancies (VP). The total occupancy of the combined site is used to detect vacancies in positionally disordered orbits. If there is more than one element occupying the orbit and the occupancy of the combined site is close to 1, then the orbit is classified as having both substitutional and positional disorder (SP). If there is more than one element occupying the orbit and the occupancy of the combined site is less than 1 minus the tolerance factor, then it is classified as having positional disorder, substitutional disorder and vacancies (SVP).

In the case when there is an external intersection of orbits (lines 9–12 of Table 1 ▸), we look at the list of elements occupying all intersecting orbits and the occupancy of the combined site. If the intersecting orbits are occupied by one element and the occupancy of the combined site is above 1 minus the tolerance factor, then the orbit is classified as purely positionally disordered (P). If the intersecting orbits are occupied by one element and the occupancy of the combined site is less than 1 minus the tolerance factor, then the orbit is classified as positionally disordered with vacancies (VP). If the list of elements on all intersecting orbits has more than one element and the occupancy of the combined site is close to 1, then the orbit is classified as having positional disorder and substitutional disorder (SP). If the list of elements on all intersecting orbits has more than one element and the occupancy of the combined site is less than 1 minus the tolerance factor, then the orbit is classified as having positional disorder, substitutional disorder and vacancies (SVP). The combined orbit produced by the external intersection of orbits *A* and *B* in Fig. 2 ▸ is an example of an SVP orbit.

In this analysis we do not distinguish between combined orbits produced via internal and external intersection, *e.g.* in Table 1 ▸ we use designations P for rows 5 and 9, VP for rows 6 and 10, SP for rows 7 and 11, and SVP for rows 8 and 12, thus defining seven distinct types of disordered orbits.

**Figure 3:**
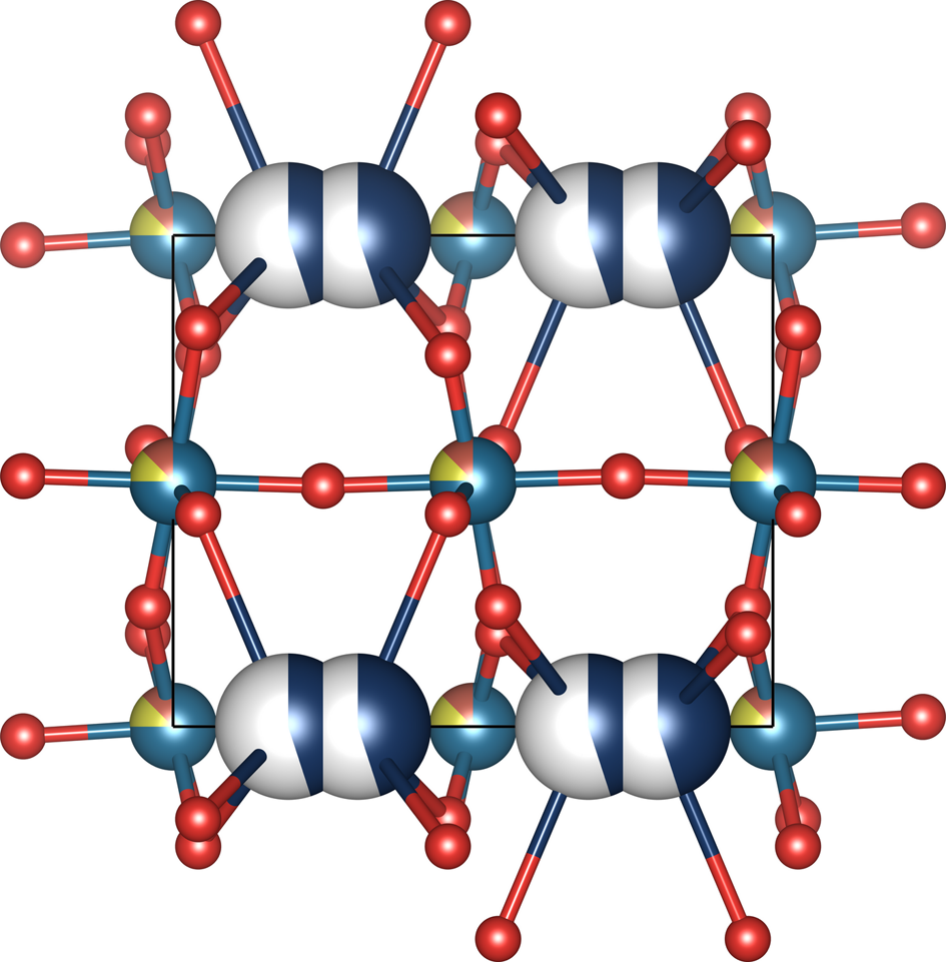
An example of a compound with a VP orbit formed by internal intersection of K sites with the occupancy of the combined site less than 1. Chemical formula K_0.3_Ta_0.125_V_0.125_W_0.75_O_3_, collection code 239274 (Rahman *et al.*, 2016 ▸). Colour code: dark blue K, brown V, yellow Ta, grey–blue W and red O.

To illustrate how the classification works, let us consider K_0.3_Ta_0.125_V_0.125_W_0.75_O_3_, collection code 239274 (Rahman *et al.*, 2016 ▸) (Fig. 3 ▸). There are four crystallographic orbits in this structure. Two of them are ordered (O) and occupied by oxygen. The third orbit is substitutionally disordered (S) as it is occupied by Ta (occupancy 0.125), V (occupancy 0.125) and W (occupancy 0.75) whose total occupancy adds up to 1. The fourth orbit is occupied by K with occupancy 0.45 and has an internal intersection of sites, which splits all K sites into pairs (dark-blue atoms in Fig. 3 ▸). There is no external intersection. The occupancy of the combined site is 0.45 × 2 = 0.90 which is less than 1, so this orbit is classified as VP. Other examples are listed in Table 2 ▸ and further details can be found in the supporting information.

### 2.3. Quantitative measures of disorder: mixing and configurational entropy, fraction of disordered sites

Next we turn to consideration of the quantitative characteristics of disorder in crystalline materials. We give a short overview of the existing approaches in this area and describe the improvements introduced in our approach.

The common way of quantifying substitutional disorder in solid solutions is the mixing entropy (Ziman, 1979 ▸; Miracle & Senkov, 2017 ▸; McCormack & Navrotsky, 2021 ▸; Krivovichev *et al.*, 2022 ▸). Recently there was an upsurge of interest in it due to the discovery of high-entropy materials. The expression for mixing entropy per site used to quantify it is (Miracle & Senkov, 2017 ▸; McCormack & Navrotsky, 2021 ▸; Krivovichev *et al.*, 2022 ▸) 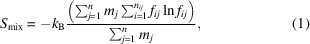
where *k*_B_ is the Boltzmann constant, *n* is the number of crystallographic orbits, *m*_*j*_ is the number of sites on the *j*th orbit (or orbit multiplicity), *n*_*ij*_ is the number of species on the *i*th sublattice of the *j*th orbit and *f*_*ij*_ are the occupancies of these species. It is assumed that 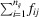 = 1 and vacancies are treated as separate species. The mixing entropy is used to classify materials with structural disorder into low-entropy (*S*_mix_ < 0.69*k*_B_), medium-entropy (0.69*k*_B_ < *S*_mix_ < 1.61*k*_B_) and high-entropy (*S*_mix_ > 1.61*k*_B_) materials (Miracle & Senkov, 2017 ▸).

Another line of research related to quantification of disorder in crystalline materials is focused on the estimation of the complexity of crystals (Krivovichev, 2016 ▸; Hornfeck, 2020 ▸; Kaußler & Kieslich, 2021 ▸; Krivovichev *et al.*, 2022 ▸). The overall idea of this approach is that the information entropy, which can be defined for both ordered and disordered mater­ials, can be used as a measure of the complexity of crystals (Krivovichev, 2016 ▸). It has been shown that in some cases information entropy can be related to the thermodynamic entropy (Krivovichev, 2016 ▸). The *CrystIT* Python package (Kaußler & Kieslich, 2021 ▸) enables the calculation of different types of information entropies and mixing entropies from CIFs. For the calculation of mixing entropy, *CrystIT* uses equation (1), assuming that only substitutional disorder and vacancies are possible. When calculating mixing entropy, vacancies are considered as additional species, so they contribute to the mixing entropy. In this paper, we distinguish between vacancies and positional disorder when interpreting partial occupancies in CIFs. By definition, positionally dis­ordered sites are located too close to each other to be occupied simultaneously, and therefore the direct use of equation (1) will overestimate the mixing entropy. To address this limitation, we consider positional disorder as the delocalization of atomic positions, which does not contribute to mixing in the sense of atomic substitutions or vacancies but increases the overall number of crystal configurations. As a result, we distinguish between mixing entropy, which accounts only for mixing between atomic species and vacancies, and configurational entropy, which accounts for all possible crystal configurations, including permutations due to mixing and contributions from positional disorder.

Note that interpreting positional disorder as the disorder of individual atomic positions is an approximation that might not always be justified. For example, observed positional disorder may in fact reflect the tilting of larger atomic groups (such as polyanions, or octahedra in perovskites), stacking disorder of entire layers *etc.* In these cases, thinking about positional disorder as the disorder (static or dynamic) of individual atomic positions leads to overestimation of the configurational entropy, because the displacements involved are locally correlated with each other rather than independent. However, as we are dealing with spatially averaged information we cannot distinguish between correlated displacements and positional disorder, and thus we state that we calculate the upper bound of the configurational entropy.

There are contributions to entropy that are beyond the scope of this paper. Specifically, the entropy of a crystalline solid apart from configurational entropy (or mixing entropy if there is no positional disorder) will also include vibrational entropy, entropy of electrons, magnetic moments and spins of nuclei. In most disordered materials, configurational and vibrational entropies dominate other terms (Miracle & Senkov, 2017 ▸). Vibrational entropy can be estimated from X-ray and neutron scattering data using atomic displacement parameters (Huang & Widom, 2022 ▸) or calculated from lattice dynamic simulation (Tolborg & Walsh, 2023 ▸). However, here we are focusing exclusively on the configurational and mixing entropies. Below we derive formulae to calculate them in the general case.

Let us first consider mixing entropy. According to Boltzmann’s formula,
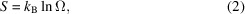
where *k*_B_ is the Boltzmann constant and Ω is the number of microstates that realize a particular macrostate. We assume that distributions of atoms on different orbits and on sites within orbits are independent of each other, such that the total entropy is the sum of the mixing entropy within the different orbits. In this case, 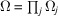 and *S*_mix_ = 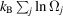 = 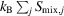. For an S orbit with index *j*, there are several species *i* which occupy it with partial occupancies *f*_*ij*_ so that 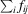 = 1. Then the number of atoms of the *i*th species on the *j*th S orbit is *m*_*j*_*f*_*ij*_, where *m*_*j*_ is the multiplicity of the orbit. The number of ways of distributing atoms over this orbit is 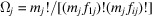, so the total entropy associated with the orbit is 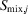 = 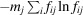 [Stirling’s formula *n*! ≃ (*n*/*e*)^*n*^ was used]. The same approach works for V and SV orbits if we consider vacancies as additional atomic species. Then per mole of atoms, for S, V and SV orbits, the mixing entropy is
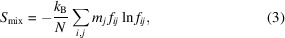
where *N* is the number of atoms in the unit cell, *m*_*j*_ is the multiplicity of the *j*th orbit, *f*_*ij*_ is the occupancy by the *i*th element of the *j*th orbit and vacancies are treated as a special type of atom in this formula (more precisely, we count their contribution to the number of permutations, but not to *N*). This formula coincides with equation (1) when there are no vacancies (the difference is only in the normalization per atom rather than per site) and can be directly applied to orbits without positional disorder.

To calculate the mixing entropy for positionally disordered orbits SP, VP and SVP we assume (i) positional disorder does not contribute to the mixing entropy, (ii) all sites within a combined site are considered as one site from the viewpoint of mixing entropy, and (iii) all occupancies related to one type of atom are summed to represent the total occupancy of the *i*th element. Then the same equation (3) can be used for the combined orbit. Let us use the SVP orbit shown in Fig. 2 ▸(*d*) as an example to explain the approach. The SVP orbit is formed by external intersection of orbits *A* and *B*. Orbit *A* is occupied by the blue element. Orbit *B* is occupied by two elements, blue and red. The multiplicity of the combined orbit, which is defined as the number of combined sites in the combined orbit, is 4. To calculate the mixing entropy, we sum all occupancies of the blue element, which gives 0.25 × 2 + 0.125 = 0.625; the occupancy for the red element is 0.125 and the occupancy for the vacancy is 0.25. Thus, the mixing entropy for this orbit is *S*_mix_ = −*k*_B_/*N* × 4 × (0.625ln0.625 + 0.125ln0.125 + 0.25ln0.25).

We can formalize the explanation in the following way: 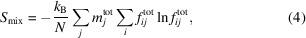
where 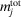 is the combined multiplicity of the *j*th orbit, 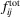 = 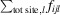 is the total occupancy for the *i*th element of the combined site, 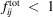, and the summation is over index *l* which labels intersecting sites within one combined site.

Note that externally intersecting orbits all have equal combined multiplicity, otherwise the entropy is not calculated.

Now we can turn to the calculation of the configurational entropy, *S*_conf_. We assume that the configurational entropy has a contribution from positional disorder, meaning that arrangements of atoms corresponding to placing atoms on different intersecting sites are calculated as separate configurations. Therefore, it is expected that the configurational entropy coincides with the mixing entropy for S, V and SV orbits, but is larger than the mixing entropy for positionally disordered orbits SP, VP, P and SVP.

The configurational entropy per individual site is 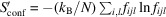, which is the well known Gibbs formula. For the orbit as a whole we need to multiply by the multiplicity of the orbit that the site belongs to. The final formula is
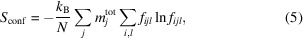
where 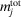 is the combined multiplicity of the *j*th orbit and *f*_*ijl*_ is the occupancy for element *i* on the combined site of the *j*th orbit, which contains *l* intersecting sites. Vacancies are considered as additional species.

Using Fig. 2 ▸(*d*) as an example of a combined orbit whose combined site comprises three crystallographic sites, and substituting the corresponding occupancies (0.25, 0.25 and 0.125 for the blue element, 0.125 for the red element, and 0.25 for a vacancy) in equation (5), we obtain *S*_conf_ = −*k*_B_/*N* × 4 × (3 × 0.25ln0.25 + 2 × 0.125ln0.125) for this orbit. Note that in this case, we have relied on the fact that all three sites forming the combined site intersect with each other, so only one atom can be placed on any of them at a time. In this scenario, the total occupancy of the combined site should not exceed 1 and equation (5) can be applied. However, there might be cases when not all sites intersect but instead they form an extended network or chain of intersecting sites where more than one atom can be placed at a time, as in Fig. S6, collection code 202039, Ca_0.82_F_2.36_Th_0.18_.

Below we consider some examples of calculating mixing and configurational entropy for compounds we considered in the previous section.

Let us consider entropy in some of our example compounds from Table 2 ▸. In Sr_2_NiN_2_ (Fig. S3) there are four orbits: (i) an ordered Sr orbit with multiplicity *m*_1_ = 4, (ii) a positionally disordered orbit occupied by Ni, which has *m*_2_ = 4 half-occupied sites that intersect and form pairs, so its combined multiplicity 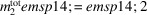, and (iii) and (iv) the last two orbits with multiplicity 4 which are positionally disordered and half-occupied by N, so their combined multiplicity 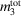 = 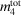 = 2. The number of atoms in the unit cell is ten. Therefore, 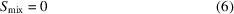
and
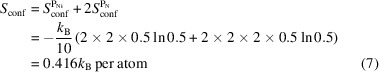
is the configurational entropy per atom, or *S*_conf_ = 2.08*k*_B_ per formula unit Sr_2_NiN_2_.

In K_0.3_Ta_0.125_V_0.125_W_0.75_O_3_ (Fig. 3 ▸) there are the following disordered orbits present: (i) a substitutionally disordered orbit S with multiplicity 6, occupied by Ta, V and W with respective occupancies 0.125, 0.125 and 0.75, and (ii) a VP orbit with internal intersection occupied by K with multiplicity *m*_*K*_ = 4 and occupancy *f*_*K*_ = 0.45, resulting in a combined site with multiplicity 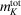 = 2 and total occupancy 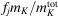 = 0.9. The number of formula units in the unit cell is *Z* = 6. Therefore, 
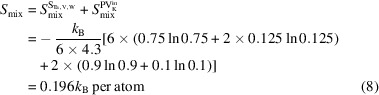
is the mixing entropy per mole of atoms and 
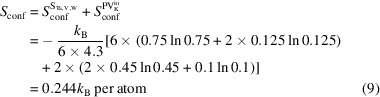
is the configuration entropy per mole. This example demonstrates that orbits without positional disorder make identical contributions to both mixing and configurational entropies, while the contribution from orbits with positional disorder to the configurational entropy is larger than that to the mixing entropy due to the contribution of positional disorder.

It is useful to introduce another quantitative measure of disorder such as the fraction of disordered sites or the fraction of sites with a particular type of disorder. This measure can be useful for the characterization of ionic conductors, for which the concentration of vacancies of mobile ions is an important parameter.

For example, for the fraction of disordered sites,
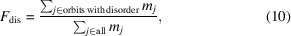
where the numerator is the summation over all orbits with disorder (of all types) and the denominator is the total number of sites. Note that if positional disorder is present the total number of sites used in this formula will be smaller than the number of sites produced from the crystallographic description in the CIF, because some sites will be combined.

### 2.4. Classification of compounds and structure types

In the previous sections we introduced a method for classifying orbits of crystalline materials according to the type of disorder and a way of quantifying the amount of disorder contained. This allows one to add a disorder description to the structure types used to describe crystalline structures in a more detailed way than specifying the symmetry, *i.e.* space group type or type of lattice. In this section, we review how the structure types are typically defined, give an example of how disorder information can be used to improve differentiation between materials within a single ICSD structure type and analyse the statistics of different orbit disorder types across the ICSD.

The definition of structure types resides on the notions of *isopointal* and *isoconfigurational* structures (Lima-De-Faria *et al.*, 1990 ▸). Two structures are *isopointal* if (i) they have the same space group type or belong to the pair of enantiomorphic space group types and (ii) the complete sequence of occupied Wyckoff positions (including the number of times each Wyckoff position is occupied) is the same for both structures when the structural data have been standardized to have the same origin and cell setting choice (because the Wyckoff letters of atomic positions can change upon a change in the origin or rotation/permutation axis). Two structures are *isoconfigurational* if (i) they are isopointal and (ii) for all corresponding Wyckoff positions both the crystallographic orbits and their geometric interrelationships are similar. Structure types are defined as groups of isoconfigurational structures. However, as *similarity* can be defined in different ways, several approaches to the definition of structure types have been developed (Mehl, 2019 ▸; Allmann & Hinek, 2007 ▸; Mehl *et al.*, 2017 ▸; Hicks *et al.*, 2019 ▸; Hicks *et al.*, 2021*a* ▸). For example, in the ICSD structure types are defined as compounds having the same space group number, similar Wyckoff sequences, similar Pearson’s symbols and similar ANX formulae, and may have additional constraints on lattice parameters, lattice angles or the types of elements which must be present or must not be present in the structure (Allmann & Hinek, 2007 ▸; Steudel *et al.*, 2025 ▸).

Since disorder is commonly not taken into account for the definition of structure types, they do not clearly distinguish between disordered and ordered structures. Taking into account that disorder is important in terms of physical properties and the number of structures containing it (approximately 50% of both the full ICSD and our reduced data set), we suggest that a disorder description could be added to definitions of structure types by modification of the Wyckoff sequence to include disorder labels. Quantitative characteristics of disorder such as configurational entropy, mixing entropy and fractions of all types of disorder can be added as additional descriptors. For example, the compound K_0.3_Ta_0.125_V_0.125_W_0.75_O_3_ (Fig. 3 ▸), collection code 239274, has space group type 182 and Wyckoff sequence 1*i* 1*h* 1*g* 1*e*. It has the structure type ‘Bronze(hex)#HTB#K0.26WO3’ which contains 18 other compounds. There are no additional parameters, such as limits on the ratio of the unit-cell parameters *etc.* for this structure type, to consider when characterizing the structure type according to the ICSD nomenclature. Eighteen out of 19 compounds in this structure type have two ordered O orbits (*h* and *i* sites), a VP orbit occupied by K (*e* site) and an orbit occupied by other metals (site *g*) which can be either substitutionally disordered, S, or ordered, O. The outlier in this set is K_0.3_W_0.9_Ta_0.1_O_3_, collection code 239259, which has a larger distance between K sites, so the orbit occupied by K is classified as V instead of VP. Therefore, the possible Wyckoff strings with disorder labels are 1*i*O 1*h*O 1*g*S 1*e*VP, 1*i*O 1*h*O 1*g*O 1*e*VP and 1*i*O 1*h*O 1*g*S 1*e*V. The possible fractions of disordered sites in the form (*F*_O_, *F*_S_, *F*_VP_, *F*_V_) (fraction of ordered sites, fraction of substitutionally disordered sites, fraction of VP sites and fraction of vacancy sites) are, respectively, (0.692, 0.2307, 0.077, 0), (0.923, 0, 0.077, 0) and (0.643, 0.214, 0, 0.143). In addition to using orbit disorder labels to differentiate between materials within a single ICSD structure type, we can quantify this difference by calculating mixing and configurational entropy contributions as described in this paper (see Section 2.3). For example, the mixing entropy of compounds in the ‘Bronze(hex)#HTB#K0.26WO3’ structure type varies in the range from 0.025*k*_B_ per atom to 0.216*k*_B_ per atom and configurational entropy in the range from 0.074*k*_B_ per atom to 0.264*k*_B_ per atom.

Structure types are one of the ways of classifying crystal structures. Focusing exclusively on disorder, we can describe a compound as a ‘bag of orbits’, where each compound is represented by the set of disorder labels (hereafter referred to as the ‘disorder set’) for all orbits. For example, K_0.3_Ta_0.125_V_0.125_W_0.75_O_3_ (Fig. 3 ▸) has two O orbits, one S orbit and one VP orbit, which means that it is characterized by the disorder set {O, S, VP}. In general, as there are eight types of orbit, O, S, P, V, SP, SV, VP and SVP, the number of different disorder sets which can be created out of them is 2^8^ − 1 = 255 (excluding the empty set). However, not all of them are equally represented in the database of inorganic crystal structures. Specifically in our data set, only 178 disorder sets contain at least one compound.

**Figure 4:**
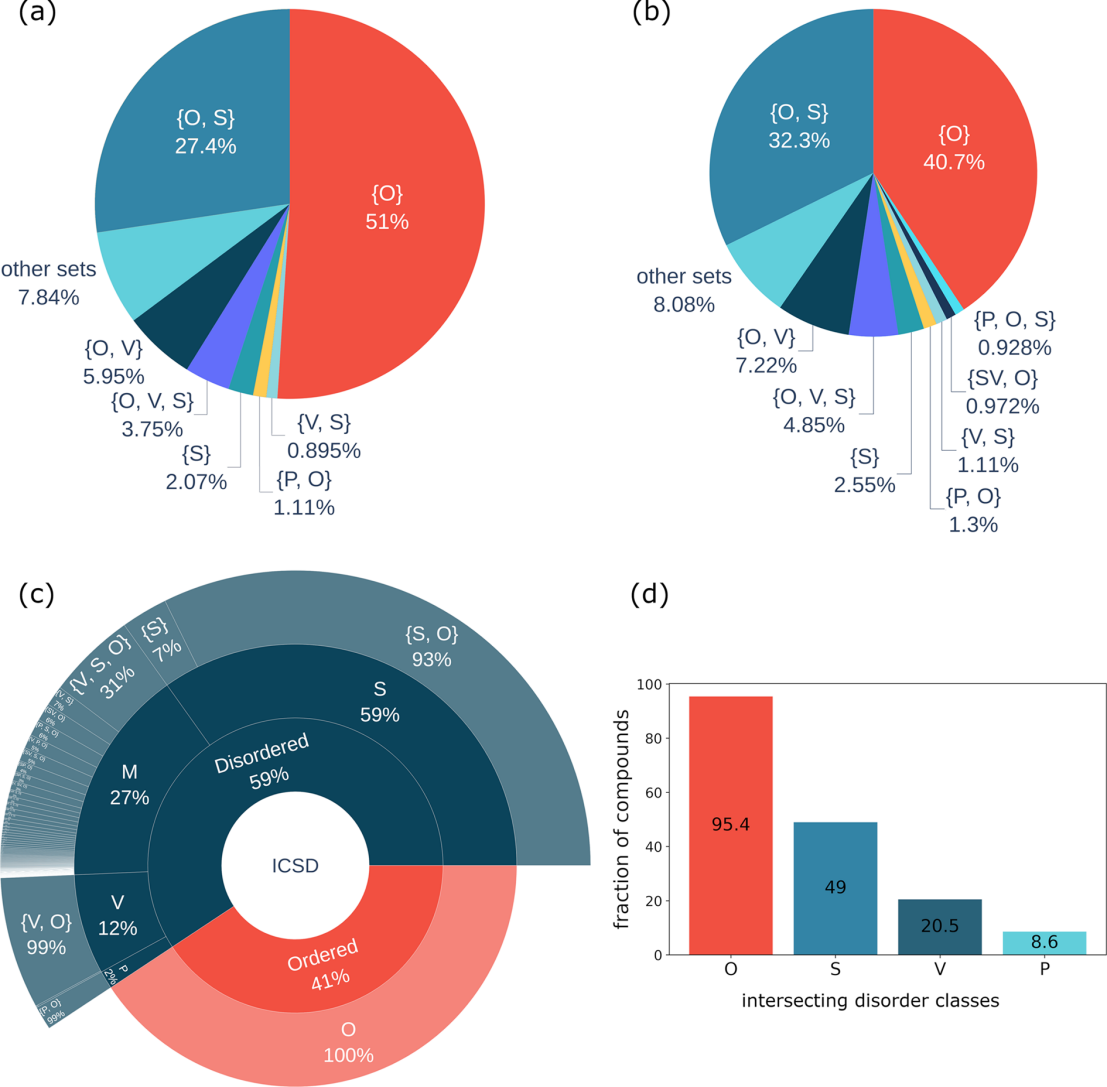
(*a*) Proportion of different types of disorder sets in the ICSD (compounds containing H are excluded). (*b*) Proportion of different types of disorder sets in the ICSD when duplicates are excluded (two compounds are considered as duplicates if they have the same composition and space group number). (*c*) Non-intersecting aggregated classes: O (set {O}), S (sets {S} and {S, O}), V (sets {V} and {V, O}), P (sets {P} and {P, O}) and M (mixed disorder set). The percentage on the plot shows the percentage of the segment with respect to the parent category. (*d*) Intersecting aggregated classes: O (all compounds which have at least one O orbit), S (at least one S, SV, SP or SVP orbit), V (at least one V, SV, VP or SVP orbit) and P (at least one P, VP, SP or SVP orbit).

Fig. 4 ▸(*a*) shows the most common types of disorder sets for compounds in our data set. One can see that 51.0% are fully ordered materials. The most common types of disorder are {O, S} 27.4%, {O, V} 5.95%, {O, V, S} 3.75%, {S} 2.07%, {P, O} 1.11% and {V, S} 0.89%. Other disorder sets are represented by less than 0.8% of entries each. The remaining 163 disorder sets have on average 67.8 compounds in them, with 87 sets containing fewer than ten ICSD entries.

The equivalent distribution [Fig. 4 ▸(*b*)] but for the data set with removed duplicates is {O} 40.7%, {O, S} 32.3%, {O, V} 7.2%, {O, V, S} 4.9%, {S} 2.6%, {P, O} 1.3% and {V, S} 1.1%. Two entries are considered to be duplicates if they have the same composition and space group. We then choose to keep only the duplicate with the highest configurational entropy. Calculation of configurational entropies is described in Section 2.3. The removal of duplicates reduces the fraction of ordered compounds from 51% to 40.7%. Removing duplicates reduces the bias in the data set that exists with respect to compounds reported multiple times. For example, there are 33 entries for CaTiO_3_ with space group 62 in our data set. The total number of disorder sets in the data set with duplicates removed remains 170, with 88 sets containing fewer than ten ICSD entries.

As the number of disorder sets is still large for practical purposes of machine learning, we continue aggregating disorder types and introduce the following classes of compounds: ordered ({O}), compounds with only substitutional disorder S = ({S}, {O, S}), compounds with disorder only due to vacancies V = ({V}, {O, V}), compounds only with positional disorder P = ({P}, {O, P}) and compounds with mixed disorder M = (all other sets). This classification creates five non-overlapping sets. The distribution over these sets is shown in Fig. 4 ▸(*c*). Ordered compounds are the most abundant at 41%, followed by compounds with only substitutional disorder, 35% (59% of all disordered compounds), only vacancies, 7.1% (12% of all disordered compounds), only positional disorder, 1.2% (2% of all disordered compounds), and mixed disorder, 16% (27% of all disordered compounds).

An alternative approach to aggregation that we considered is to create overlapping sets by putting into the O class all compounds which have at least one ordered orbit, into the S class all compounds containing any sort of substitutional disorder, into the V class all compounds containing any sort of vacancies and into the P class all compounds containing any sort of positional disorder. The results of this type of aggregation are shown in Fig. 4 ▸(*d*). We can see that 95.4% of compounds have at least one ordered orbit, 49.0% have substitutional disorder, 20.5% have vacancies and only 8.6% have any type of positional disorder.

## 3. Results and discussion

### 3.1. Distribution of structural disorder in the ICSD

#### 3.1.1. Global order/disorder in materials

Initially, we examine the distribution of order and disorder across the ICSD, just splitting them into ordered and dis­ordered.

**Figure 5:**
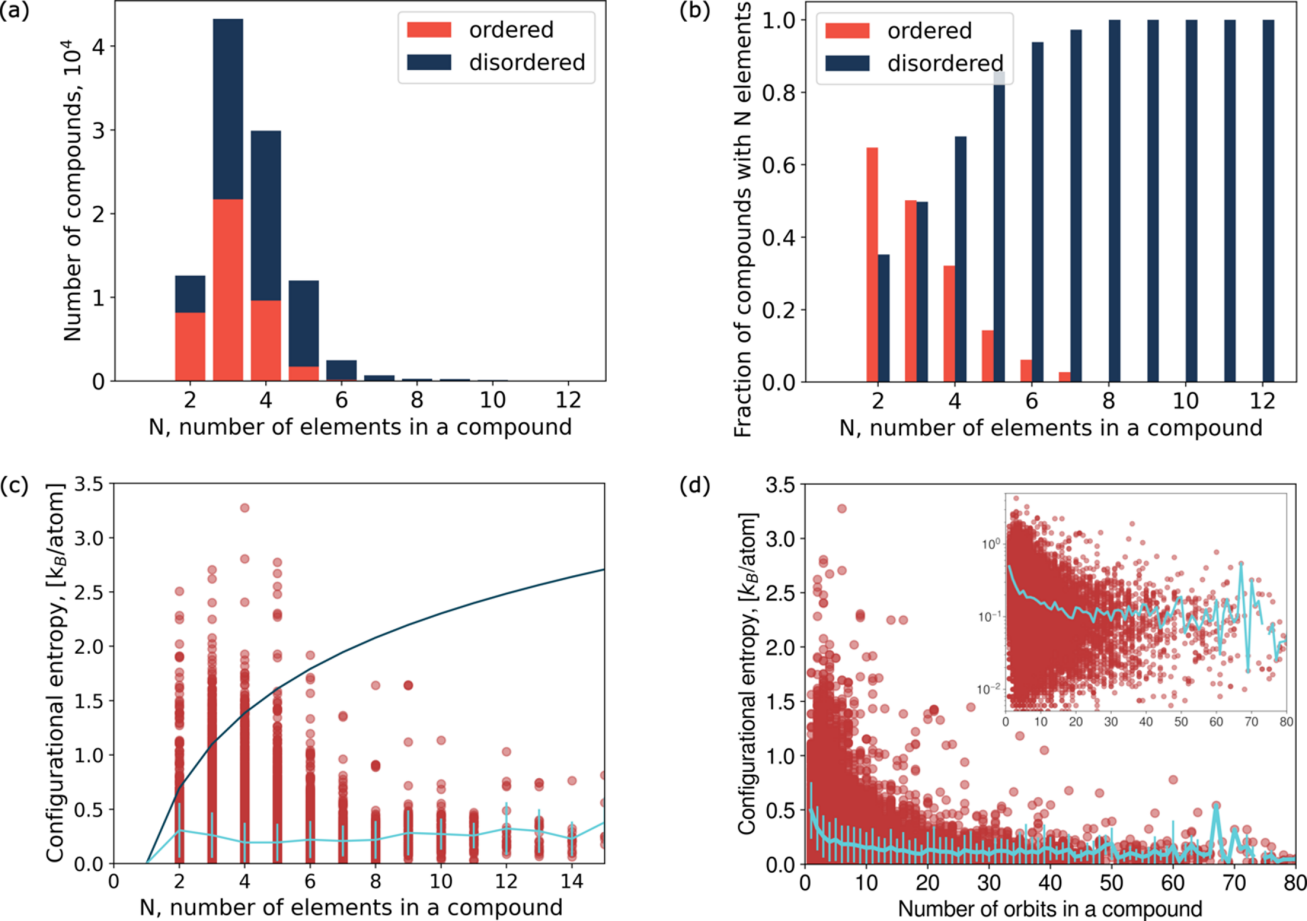
(*a*) Distribution of compounds with respect to the number of elements in a composition formula. (*b*) Proportion of ordered and disordered compounds depending on the number of elements in a formula. (*c*) Distribution of configurational entropies for compounds with different numbers of elements in their composition. Red dots represent individual compounds. The solid dark-blue line shows 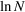 scaling and the light-blue line shows the average configurational entropy dependence on the number of elements in the compound. (*d*) Distribution of configurational entropies for compounds with different numbers of orbits in the structure. The inset shows the same plot with a logarithmic *y* axis. The light-blue lines show the average entropy dependence on the number of orbits. Red dots show individual compounds.

Fig. 5 ▸(*a*) shows the dependence of the distribution of the compounds in the ICSD with respect to the number of elements in the formula (here and below, if not specified separately, we use the data set with excluded duplicates; see Section 2 for the definition of duplicates). Compounds composed of two, three, four or five elements dominate. Fig. 5 ▸(*b*) shows the proportion of ordered and disordered compounds depending on the number of elements in the formula. The general trend coincides with the observations of Toher *et al.* (2019 ▸) in that, as the number of elements in the formula increases, the proportion of ordered compounds decreases. Interestingly, already for three-element com­pounds, the number of ordered and disordered compounds is approximately the same, and the number of disordered four-element compounds exceeds the number of ordered four-element compounds. All compounds with 11 or more elements in the formula are disordered.

Toher *et al.* (2019 ▸) showed that the enthalpy gain for the formation of ordered materials decreases very fast with increasing number of elements *N* in the compound and is exceeded by the entropy gain already for *N* = 3. Here, the enthalpy gain, Δ*H*(*N* | {1,…, *N* − 1}), of an ordered com­pound with *N* elements in its composition formula with respect to all possible ordered {1,…, *N* − 1} sub-components is defined as the energetic distance of the *N*-compound from the convex hull formed by the {1,…, *N* − 1} sub-components. The estimation of the entropy gain is based on the assumption that every site can be occupied by any of the *N* elements, so the entropy per atom is 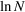 and the entropy gain is 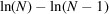.

Fig. 5 ▸(*c*) shows the dependence of the configurational entropy per atom on the number of elements in a compound [equation (5)]. The entropy is measured in units of *k*_B_ per atom. At *T* = 300 K, *S*_conf_ = 1*k*_B_ per atom corresponds to *S*_conf_*T* = 26 meV per atom. Entropies calculated from ICSD CIFs as described in Section 2.3 were used. The red dots in Fig. 5 ▸(*c*) correspond to individual compounds, the light-blue line shows the average value of the entropy for compounds with a fixed number of elements, and the standard deviations are also shown. The dark-blue line shows the 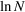 trend as a reference for systems like equimolar high-entropy alloys in which every site can be equally occupied by any of *N* elements. The range of configurational entropies per atom does not increase substantially with *N* (for *N* > 2) and the average configurational entropy grows only slightly as *N* increases. This implies that, on average, if we increase the number of elements in a compound, its complexity measured by the number of orbits increases, instead of mixing elements to a greater extent in one orbit.

Fig. 5 ▸(*d*) shows the dependence of configurational entropy on the number of orbits for all compounds in the data set. The inset shows the same plot with a logarithmic *y* axis. The average configurational entropy per atom decreases with increasing number of orbits. The number of orbits can be used as a simple measure of structural complexity, so we can state that more structurally complex compounds tend to have smaller levels of disorder than more structurally simple ones.

**Figure 6:**
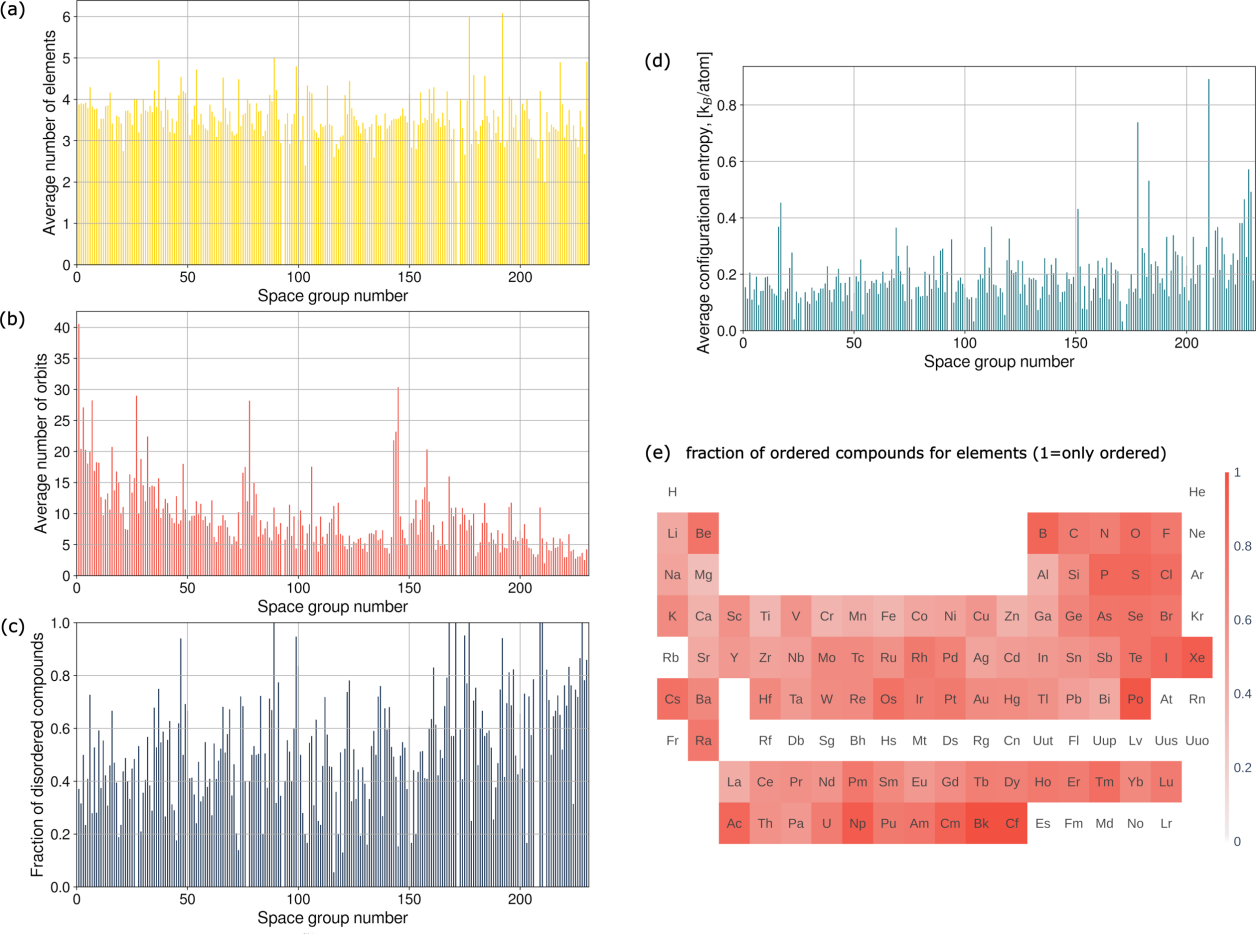
(*a*) Dependence of the average number of elements in a composition on the space group number. (*b*) Dependence of the average number of orbits in a structure on the space group number. (*c*) Dependence of the fraction of disordered compounds on the space group number. (*d*) Dependence of the average configurational entropy (averaged only over disordered compounds) on the space group number. (*e*) Fraction of compounds for each element in which it occupies an ordered orbit.

Figs. 6 ▸(*a*) and 6 ▸(*b*) show the average number of elements and orbits for each space group. The average number of elements does not depend on the space group number, but the average number of orbits decreases noticeably with increasing space group number. Fig. 6 ▸(*c*) shows the dependence of the fraction of disordered compounds on the space group number. On average, the fraction of disordered compounds increases with increasing space group number. The degree of disorder of disordered compounds measured by the average configurational entropy also increases with increasing space group number [Fig. 6 ▸(*d*)].

Fig. 6 ▸(*e*) shows for each element the fraction of compounds in which this element occupies an ordered orbit. On average, the fraction of disordered compounds decreases with increasing atomic number and with the period in each group. Non-metals are in general more ordered than metals.

#### 3.1.2. Distribution of different types of disorder over elements and structures

**Figure 7:**
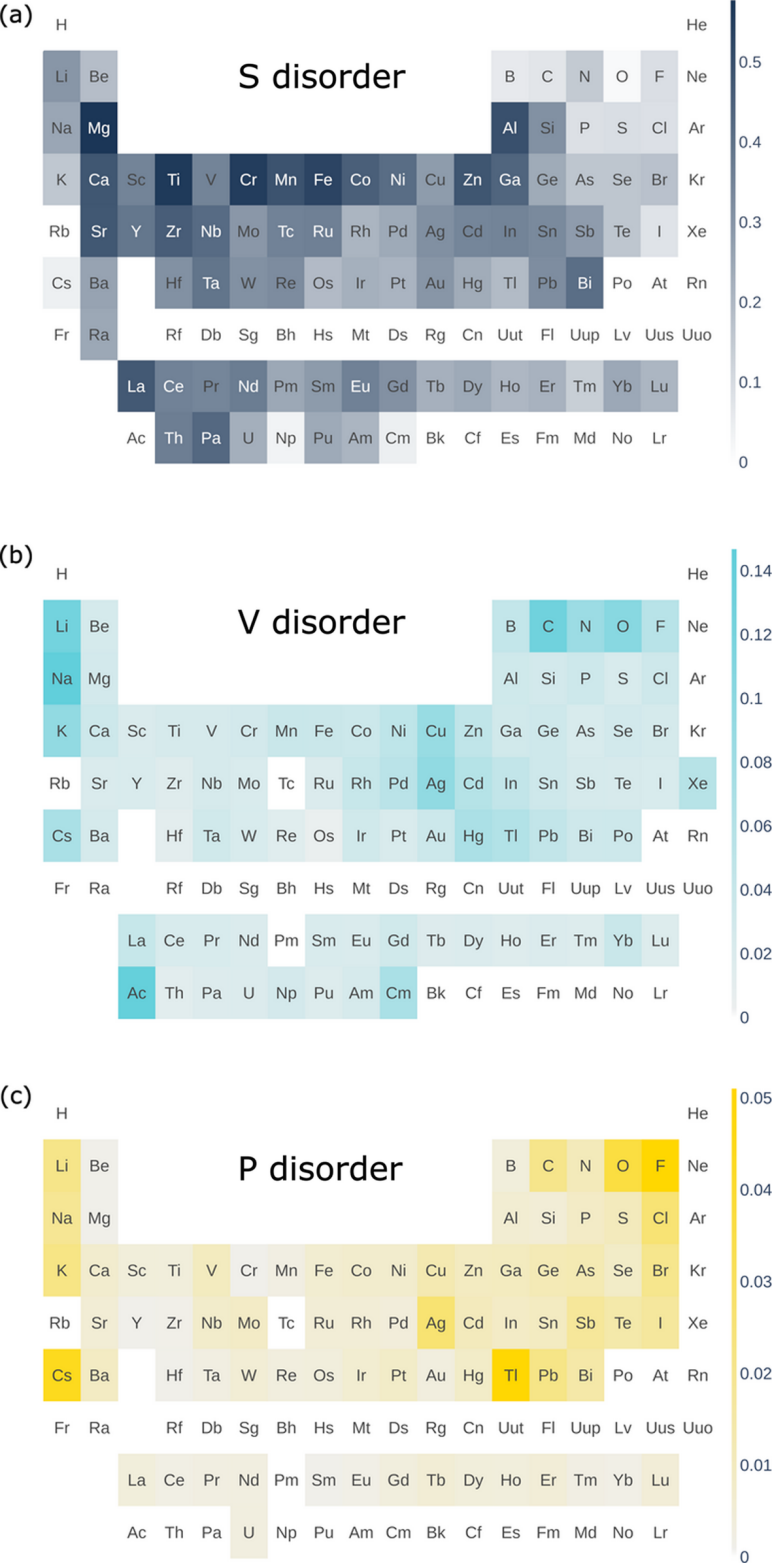
Fractions of compounds in which an element occupies (*a*) a substitutionally disordered orbit S, (*b*) an orbit with a vacancy V and (*c*) an orbit with simple positional disorder P among all compounds containing this element. Similar diagrams for other types of disorder SV, SP, VP and SVP can be found in the supporting information.

Now we turn to the distribution of different types of disorder over elements. Fig. 7 ▸ shows the fraction of compounds in which an element occupies S, V and P orbits among all compounds containing this element. The supporting information contains maps for the other types of orbits, SV, SP, VP and SVP.

The counts are aggregated in the following way. For each element the compound is counted once for each type of disorder. For example, in RbCr(SO_4_)_2_ (Fig. S8), collection code 173671, oxygen occupies both one ordered orbit and one positionally disordered orbit (in this sense we use the word ‘orbit’ according to our classification). Therefore, for this compound one count will be added to the number of compounds with an ordered oxygen orbit, and one count will be added to the number of compounds with a positionally disordered orbit. In Ca_0.97_Co_0.199_Mg_0.831_Si_2_O_6_ (Fig. S9), collection code 74470, there are three ordered orbits for oxygen. Therefore, one count will be added to the list of ordered compounds for oxygen.

Some trends can be noticed in Fig. 7 ▸. The most common type of disorder is substitutional [this is also reflected in Figs. 4 ▸(*b*) and 4 ▸(*c*) showing the distribution of compounds over disorder sets]. The fraction of substitutional disorder for metals has a tendency to decrease when going down the group, and from left to right in the period, for transition metals and lanthanoids. Mg, Fe, Cr and Ti have more than 0.5 probability of being found in substitutionally disordered orbits. The two largest metal atoms, Cs and Rb, have the smallest probability, below 0.07, of being found in substitutionally disordered orbits among all metals with sufficient statistics.

Vacancies are a much less common type of disorder than substitution. Alkali metals and Cu, Ag, Hg and Tl are most inclined to form vacancies among all metals. C, N and O also have a higher probability for vacancy formation than other elements on average.

Positional disorder is also most common for alkali metals and for Ag, Tl, Pb and Bi. Interestingly V, Nb, Mo and Sb have a slightly higher probability of being found in positionally disordered orbits than other elements. Among non-metal elements, F has the highest fraction of positional disorder, followed by O, Cl and C.

**Figure 8:**
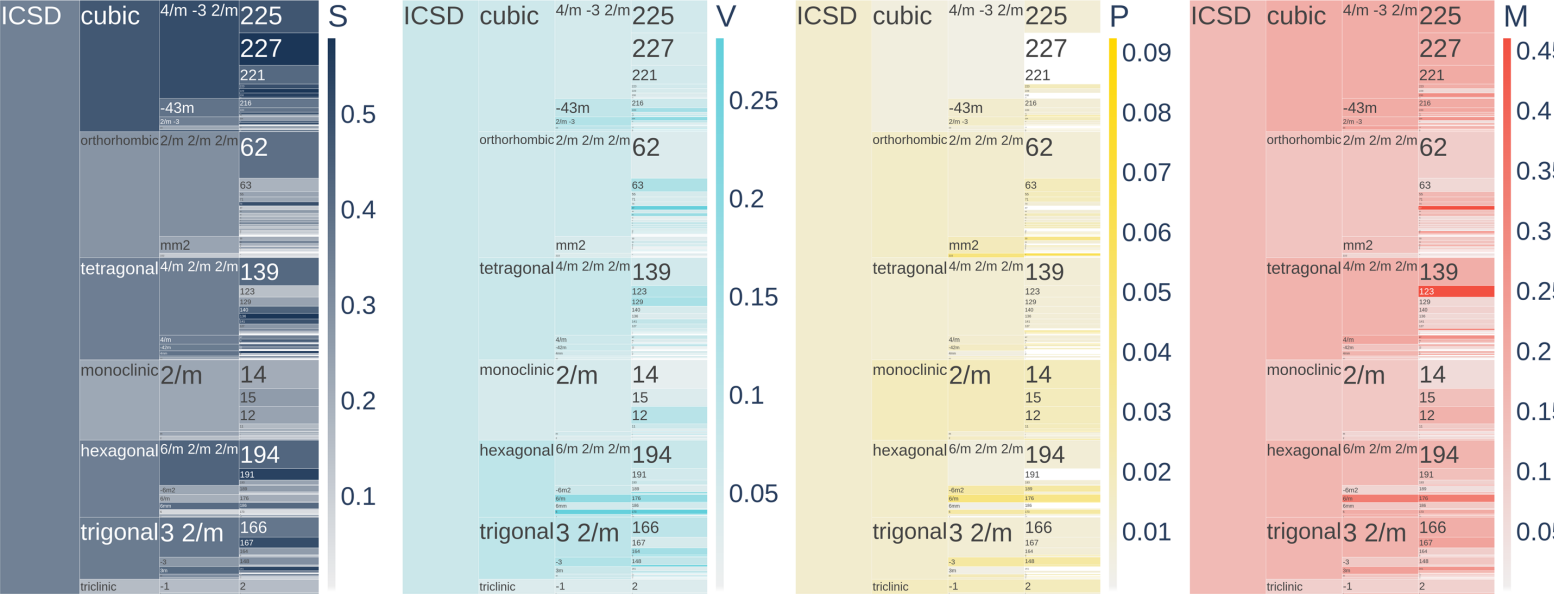
Frequency of different classes of disorder, S, V, P and M [as they are defined in Fig. 4 ▸(*c*)], over crystal systems, crystal classes and space groups. Each element of the plot is proportional in size to the number of compounds representing it, and the shading shows the fraction of the corresponding type of disorder, paler being a low fraction and darker being a higher fraction.

Fig. 8 ▸ shows the distribution of different types of disorder over structures, aggregated into crystal systems, crystal classes and space group numbers. Here we show aggregated disorder classes as they are defined in Fig. 4 ▸(*c*): S contains compounds with disorder sets {S} and {S, O}, V contains compounds with disorder sets {V} and {V, O}, P contains compounds with disorder sets {P} and {P, O}, and other compounds are aggregated into the M disorder class. In Fig. 8 ▸, each element of the plot is proportional in size to the number of compounds representing it, and the shading shows the fraction of the corresponding type of disorder.

Substitutional disorder is least common in the triclinic crystal system and most common in the cubic crystal system. Among the most populated crystal classes, 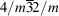 has the highest level of substitutional disorder, and the most populated space group type with the highest fraction of substitutional disorder is 227, 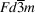. Vacancies are slightly more common in trigonal and hexagonal crystal systems than in other crystal systems, and about half as common in the triclinic crystal system. Crystal classes 6 and 6/*m* have the highest fractions of vacancies among crystal classes. In contrast to these trends, positional disorder is most common in the triclinic crystal system. However, among point groups 6/*m* and 222 have the highest fraction of positional disorder. Mixed disorder is slightly more common in the cubic and tetragonal crystal systems than in the others. Among point groups, 6/*m* has the highest fraction of mixed disorder.

### 3.2. Distribution of structural disorder in different types of materials

**Figure 9:**
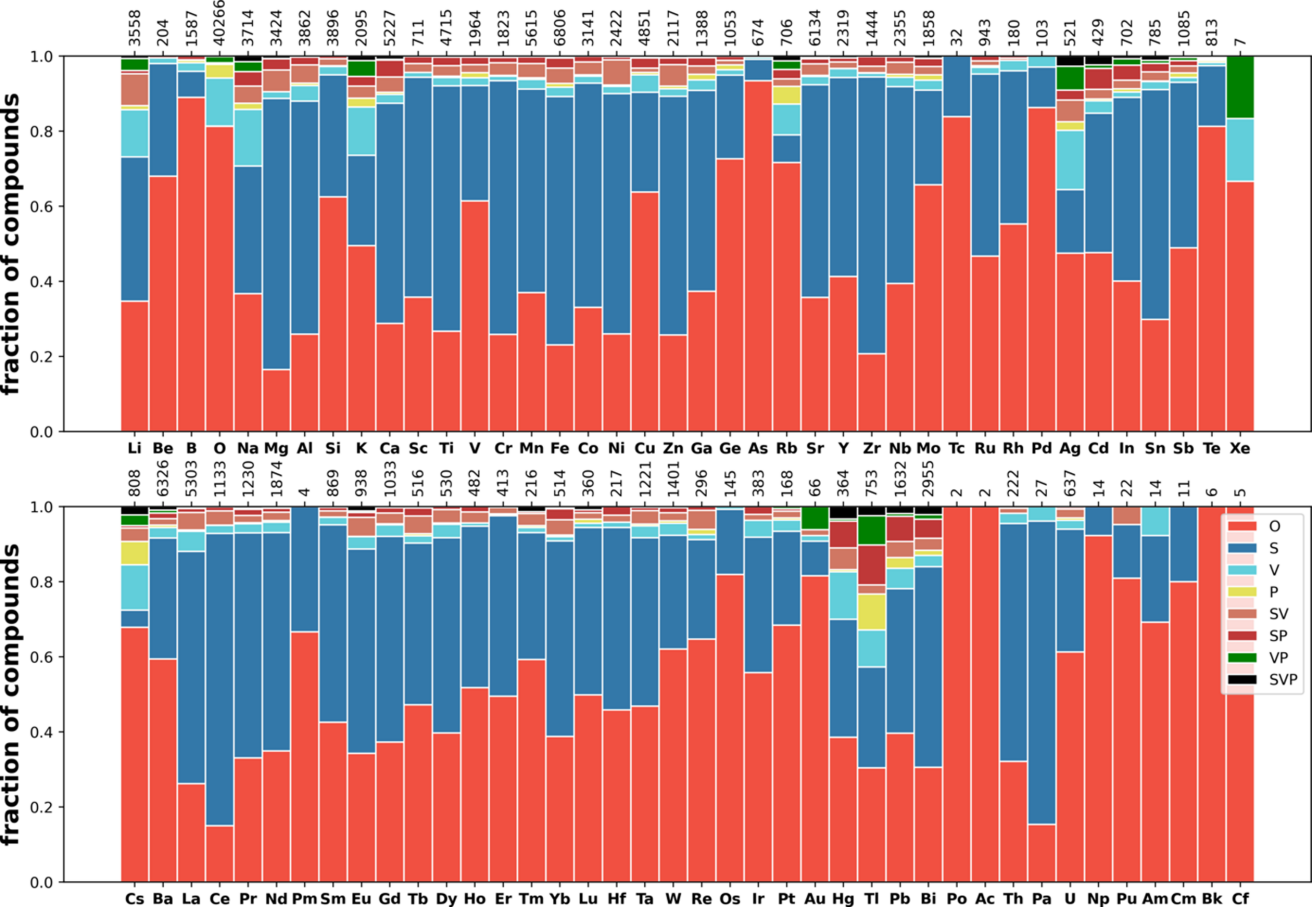
Distribution of disorder over elements in oxides.

Next we consider the distribution of disorder in several classes of materials (see Section 2.1 for the definitions of the classes) such as oxides (35098 compounds), carbides (1978 compounds), nitrides (1485 compounds), fluorides (2088 com­pounds), phosphides (1888 compounds), sulfides (5728 com­pounds), chlorides (1121 compounds), selenides (3793 com­pounds), bromides (644 compounds), iodides (658 com­pounds) and intermetallics (17766 compounds). The corresponding distributions over the elements are shown in Figs. 9 ▸–13 ▸ ▸ ▸ ▸, and Figs. S21–S26.

**Figure 11:**
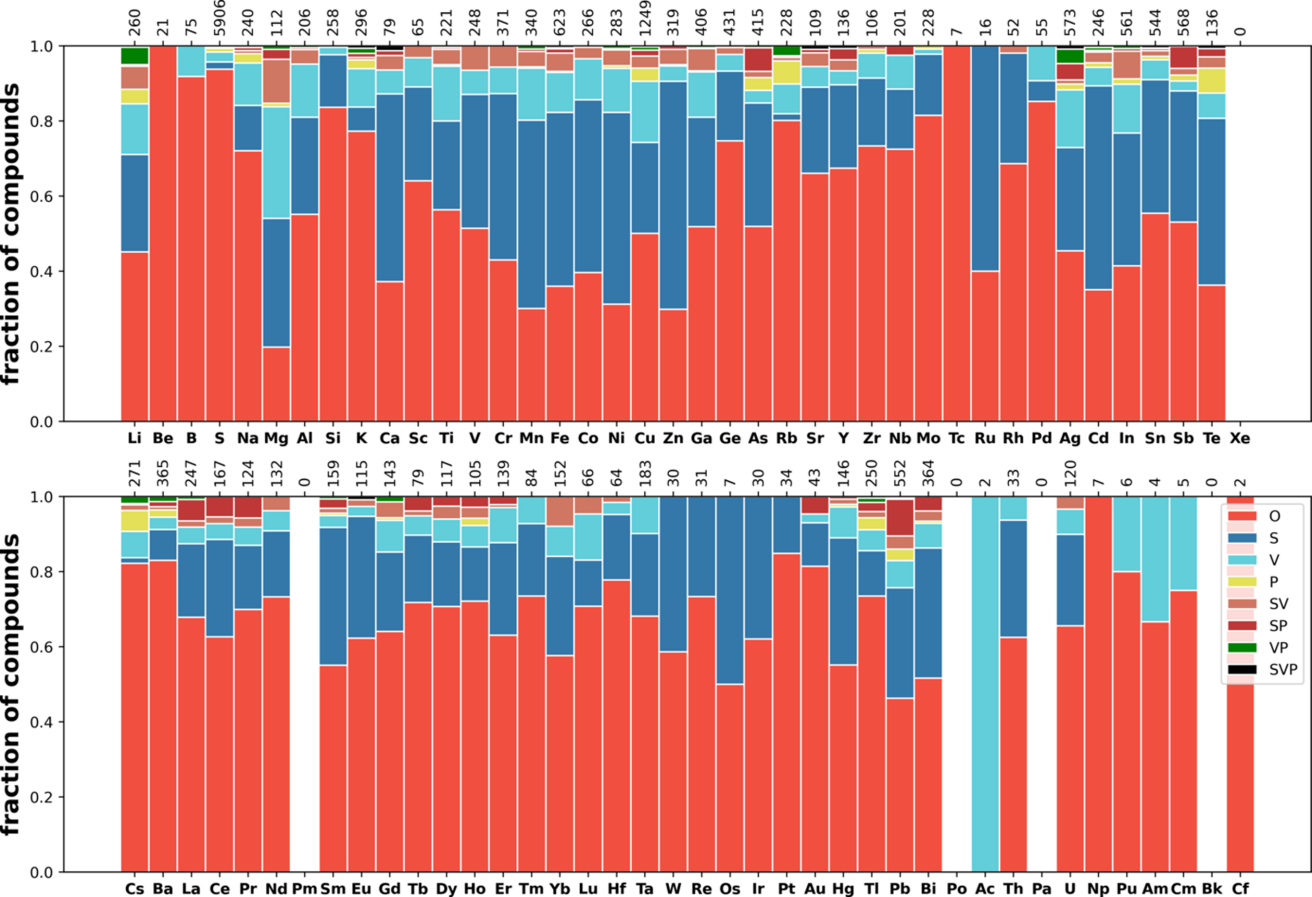
Distribution of disorder over elements in sulfides.

These distributions differ significantly from each other. Iodides and bromides have small fractions of disorder overall, and substitutional disorder is rare compared with its frequency in the database overall. In oxides (Fig. 9 ▸), metals have high fractions of substitutional disorder, including S, SV and SP. Significant fractions of vacancies are observed for alkali metals and Ag, Hg and Tl. The same elements show large fractions of positional disorder. In contrast to this picture, in sulfides (Fig. 11 ▸) and selenides (Fig. S25) nearly all metals demonstrate relatively high fractions of vacancies, including transition metals where vacancies are relatively uncommon in oxides. Phosphides, nitrides and carbides have similar distributions among themselves, with average levels of disorder. In fluorides, vacancies mixed with another type of disorder (VP, SV and SVP orbits) are nearly absent, in contrast to all other types of material. The overall fraction of disordered fluorides is small compared with oxides. Chlorides have a similar distribution to fluorides but simple substitutional disorder in chlorides is less common, and instead V and SV orbits appear.

**Figure 13:**
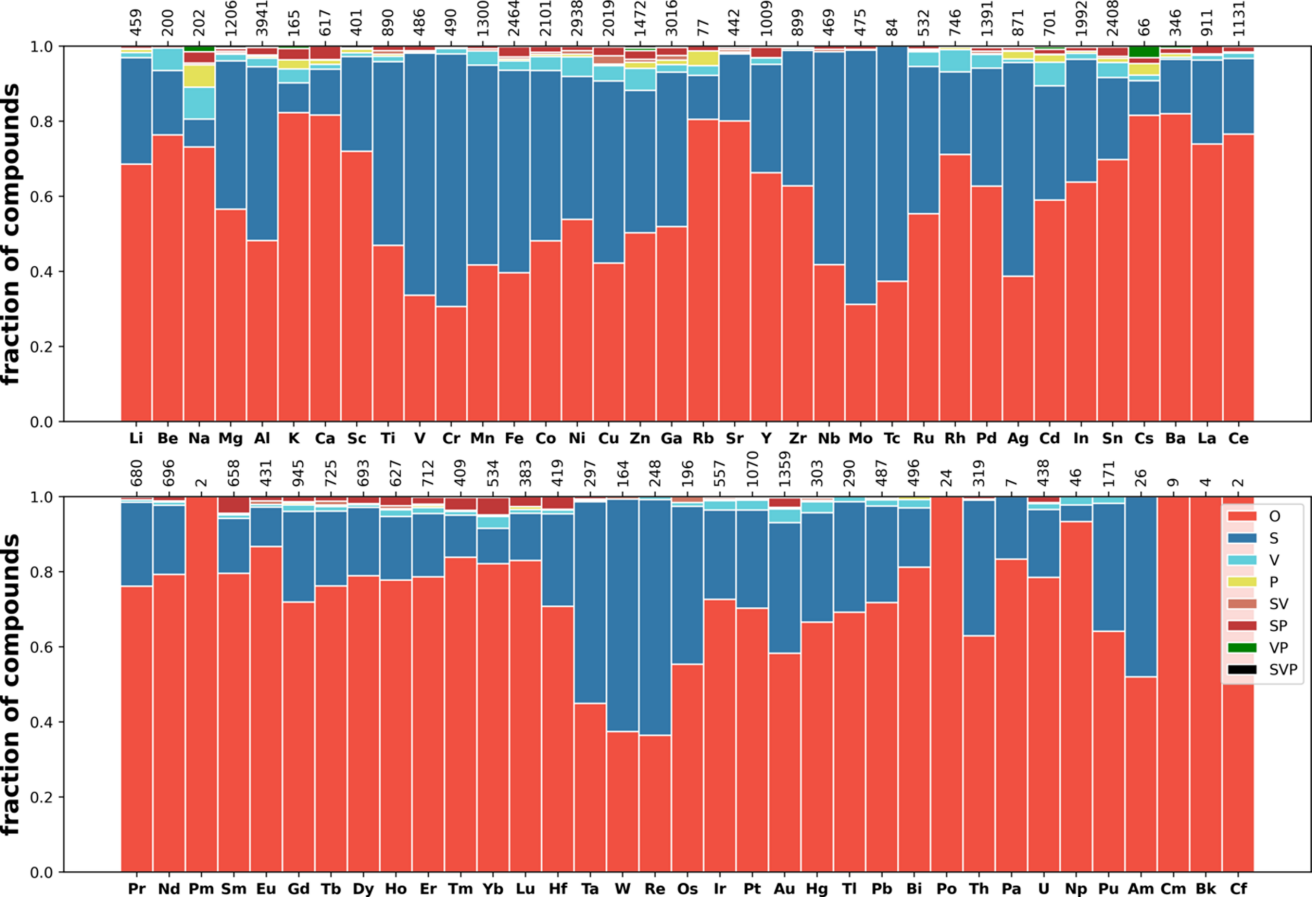
Distribution of disorder over elements in intermetallics and alloys.

Fig. 13 ▸ shows the distribution of disorder in intermetallics and alloys. In the intermetallics/alloys data set (described in Section 2.1), the fraction of disorder is on average low and predominately represented as substitutional disorder, with a small fraction of vacancies. In oxides and sulfides, alkali metals and Ag, Tl, Pb and Bi show rich disorder behaviour. In intermetallics/alloys they do not differ qualitatively from other metals.

### 3.3. Substitutional disorder

In this section we consider substitutional disorder and the probability of two elements occupying the same orbit simultaneously (*i.e.* of substituting for each other). It is interesting to compare these probabilities with the closeness of elements with respect to the Pettifor scale (Pettifor, 1984 ▸; Glawe *et al.*, 2016 ▸), which is a one-dimensional ordering of elements based on their ability to substitute each other in *ordered* compounds without changing the structure type.

**Figure 14:**
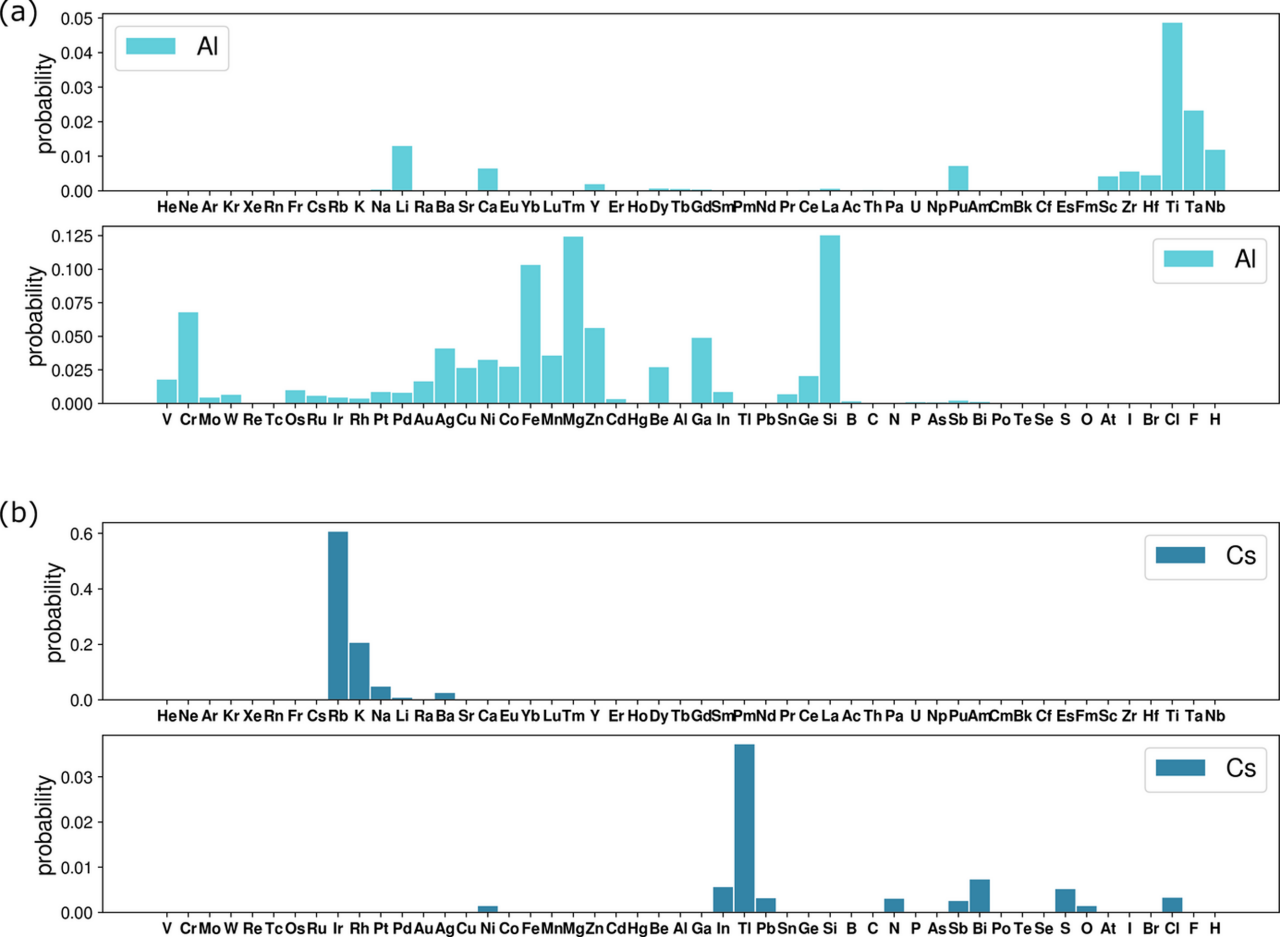
(*a*) Distribution of probabilities of finding Al and another element in one substitutionally disordered orbit. (*b*) Distribution of probabilities of finding Cs and another element in one substitutionally disordered orbit.

To do this, first we collect statistics of co-occurrence of elements in all types of substitutionally disordered orbits, S, SV, SP and SVP. We do so by counting for each element the number of times these elements appear together in one orbit, *O*_*ij*_(S, SV, SP, SVP). To quantify the difference in frequency of occurrence of elements in reported materials, we divide the count by the number of compounds with element *i*, *N*_*i*_, and element *j*, *N*_*j*_:
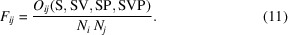
Then for each element we sum the adjusted counts [equation (11)] over all other elements and divide each count by this number:
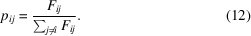
As a result, for each element *i* we have the list of probabilities, *p*_*ij*_, for this element to be found together with another element *j* in a substitutionally disordered orbit. Examples of such lists of probabilities are shown in Fig. 14 ▸(*a*) for Al and Fig. 14 ▸(*b*) for Cs. To facilitate comparison with the Pettifor scale, the elements on the *x* axes of Figs. 14 ▸(*a*) and 14 ▸(*b*) are ordered according to the modified Pettifor scale (Glawe *et al.*, 2016 ▸). The highest probability of substituting Cs is for Rb and K, which are the closest to Cs on the Pettifor scale. Al has the highest probability of being substituted by Si, Mg, Fe, Cr and Ti, which are all a long way from Al on the Pettifor scale. Thus, the Pettifor number difference is a good predictor of substitution for Cs, but not for Al.

**Figure 15:**
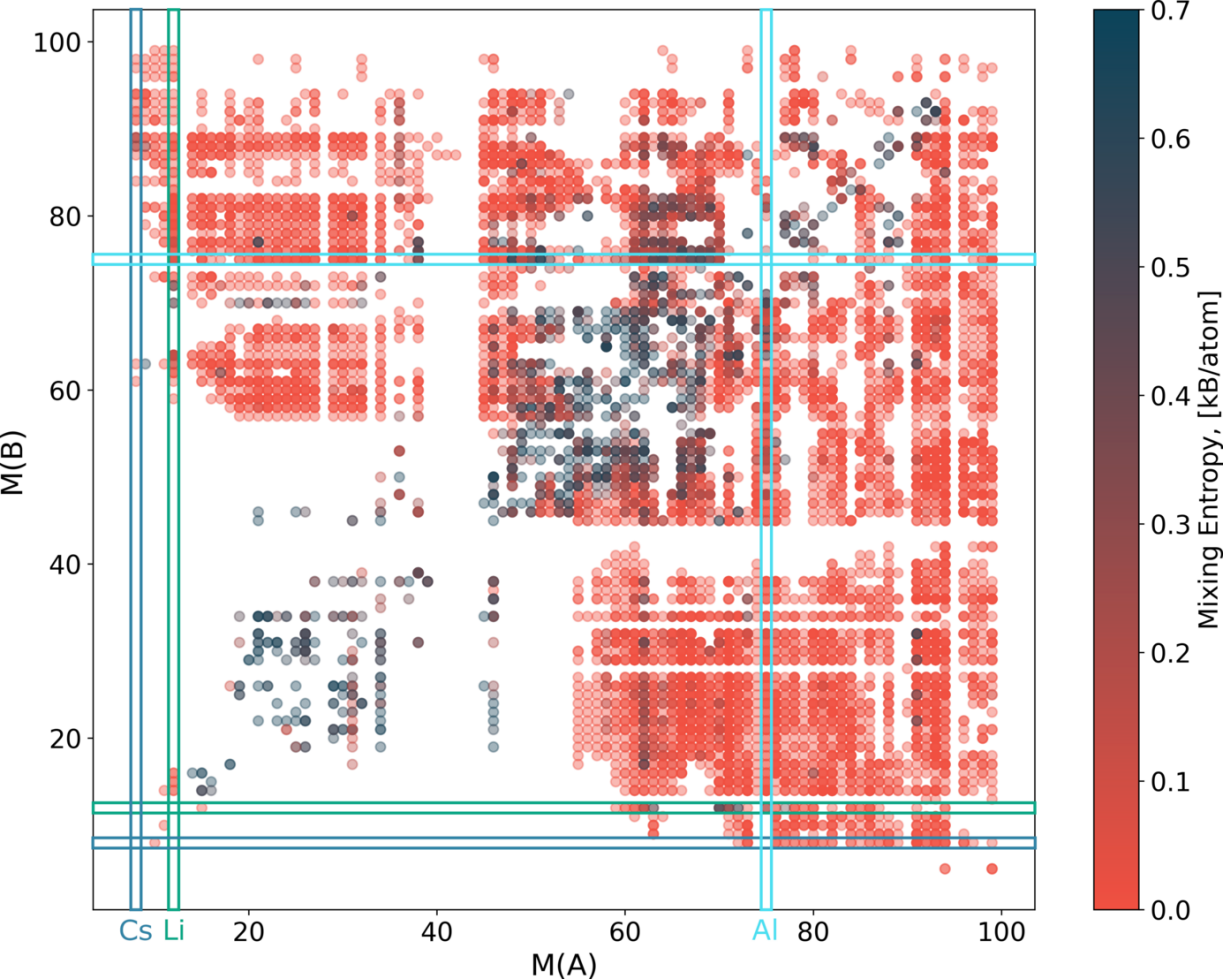
Pettifor number *M*–*M* plot for binary compounds *A*_*a*_*B*_*b*_. The convention is that *a* ≥ *b*. Only ordered compounds and compounds with substitutional disorder are shown. The colour represents the value of the mixing entropy, dark blue corresponding to the maximum and red to the minimum.

Fig. 15 ▸ shows an *M*–*M* plot for binary compounds *A*_*a*_*B*_*b*_, *a* > *b*, which are ordered or have substitutional disorder only. *M*(*A*) is the Pettifor number for element *A* and *M*(*B*) is the Pettifor number of element *B*. Each circle represents a compound. The colour of the circle reflects the value of the mixing entropy per atom in the compound. The maximum entropy corresponds to the ideal mixing of two elements on one site, 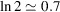 (occupancies of 0.5 for both elements). Compounds with maximum mixing entropy tend to cluster along the diagonal, meaning that binary compounds composed of elements with similar Pettifor numbers tend to form disordered compounds. In contrast, compounds with a large difference in Pettifor numbers tend to form ordered compounds. However, there are some elements (such as Li and Al) for which this rule does not work, even in binary compounds.

In conclusion, we can say that the difference in Pettifor numbers correlates, on average, with the probability of elements substituting each other in substitutionally disordered orbits. Elements with close Pettifor numbers tend to have a high degree of mixing, measured by the mixing entropy, but this is not true for all elements.

## 4. Conclusions

In this work we have analysed the distribution of disorder in the Inorganic Crystal Structure Database. To facilitate our analysis, we devised a classification system for crystallographic orbits concerning structural disorder. We identified seven distinct types of disordered orbits, S, V, P, SV, SP, VP and SVP. The tools which we have developed can be used to identify the type of disorder present in experimental CIFs.

Current approaches to the definition of crystallographic structure types do not take disorder into account. As approximately 50% of known compounds contain disorder, we suggest the addition of disorder descriptors to the definition of structure types and we take a first step in this direction. A reliable definition of structure types requires developing robust measures of similarity, taking into account both geometric, elemental and disorder information, which is beyond the present work.

We have demonstrated that, focusing solely on disorder, compounds can be categorized according to the combination of disordered orbit types present in their structures. In our data set, there are 170 such disorder sets, with five of them encompassing over 90% of the structures, {O}, {S, O}, {V, O}, {S, V, O} and {S}. Continuing a coarse-graining of representations of compounds, we can divide the compounds into a smaller number of classes: for example, ordered compounds, or compounds with substitutional disorder, positional disorder, vacancies or mixed disorder. The classification can be adjusted for each particular purpose.

To quantify disorder we calculate the mixing and configurational entropy from the CIFs. To do this we build on a recently implemented method for entropy calculation (Kaußler & Kieslich, 2021 ▸), but in the present paper we distinguish between vacancies and positional disorder, which requires new formulae for the calculation of those entropies. In particular, we recognize the fact that positional disorder does not contribute to the mixing entropy but contributes to configurational entropy. We derive new formulae here.

The notions of ‘combined orbit’ and ‘combined site’ which we have introduced allow the determination of average coordination environments for positionally disordered sites when the coordination environment can be determined for the centre of mass of a ‘combined site’. At the moment, existing packages for the determination of local coordination environments (Waroquiers *et al.*, 2020 ▸) cannot correctly process such cases. We do not address this question in the present paper, but it may be done in the future.

We have analysed the distribution of disorder over elements and structures. Substitutional disorder is much more common than any other type of disorder. On average, compounds in higher-number space groups tend to be more disordered (Fig. 6 ▸). These more symmetric structures, on average, have a smaller number of orbits but they tend to have higher configurational entropy. The average configurational entropy per atom grows very slowly with increasing number of elements in the composition, in contrast to previous predictions (Toher *et al.*, 2019 ▸). At the same time, the configurational entropy per atom decreases with increasing number of orbits.

The distribution of disorder over the elements has noticeable trends. The fraction of disorder decreases with increasing atomic number, down the group for all metals. The fraction of disorder also decreases with increasing atomic number for transition metals, lanthanoids, actinoids and lighter *p*-block metals. Tl, Pb and Bi show the opposite trend. Alkali metals and Ag, Tl, Pb and Bi have much larger fractions of positional disorder, vacancies and complex disorder types than other metals, on average.

We have also analysed the distribution of disorder in different classes of compounds and find some clear trends. For example, fluorides are largely free of vacancies, whereas sulfides have a relatively high percentage of compounds with vacancies. Bromides and iodides have low levels of disorder in general. In intermetallics substitutional disorder is common, while other types of disorder are not common. Alkali metals and Ag, Tl, Pb and Bi do not show unusual behaviour in intermetallics and behave like all other metals.

Finally, we looked more attentively at substitutional disorder. The question we asked was whether the Pettifor number is a good predictor of the probability of two elements being present in one substitutionally disordered orbit. The Pettifor number was derived as a measure of the probability of two elements substituting each other in an ordered compound without changing the structure type. The answer is that the difference in Pettifor numbers of two elements is a good measure of the probability that they share the same substitutionally disordered orbit on average, but some elements deviate strongly from this rule.

We have introduced a classification of disorder in crystals described by the average structure and developed a program performing automatic classification. This may have a variety of benefits, as 50% of experimentally discovered crystal structures available in the ICSD contain disorder. For example, disorder could be introduced into the existing classifications of crystal structures, or the introduction of disorder descriptors may facilitate high-throughput and machine learning studies of disordered materials.

## 5. Code availability

The code for entropy calculation from a CIF and the code used to generate all images are available at https://github.com/lrcfmd/Disorder.

## 6. Related literature

For further literature related to the supporting information, see Batuk *et al.* (2017 ▸), Brauer & Gradinger (1954 ▸), Brown (2009 ▸), Guymont *et al.* (1992 ▸), Hellmann & Mewis (1950 ▸), Kim *et al.* (2019 ▸), Kowach *et al.* (2000 ▸), Laval *et al.* (1986 ▸), Leube *et al.* (2022 ▸), Malaman *et al.* (1976 ▸), Shannon & Prewitt (1969 ▸), Tabira *et al.* (1992 ▸) and West *et al.* (2008 ▸).

## Supplementary Material

Additional discussion and examples. DOI: 10.1107/S1600576725003000/jur5002sup1.pdf

## Figures and Tables

**Figure 10:**
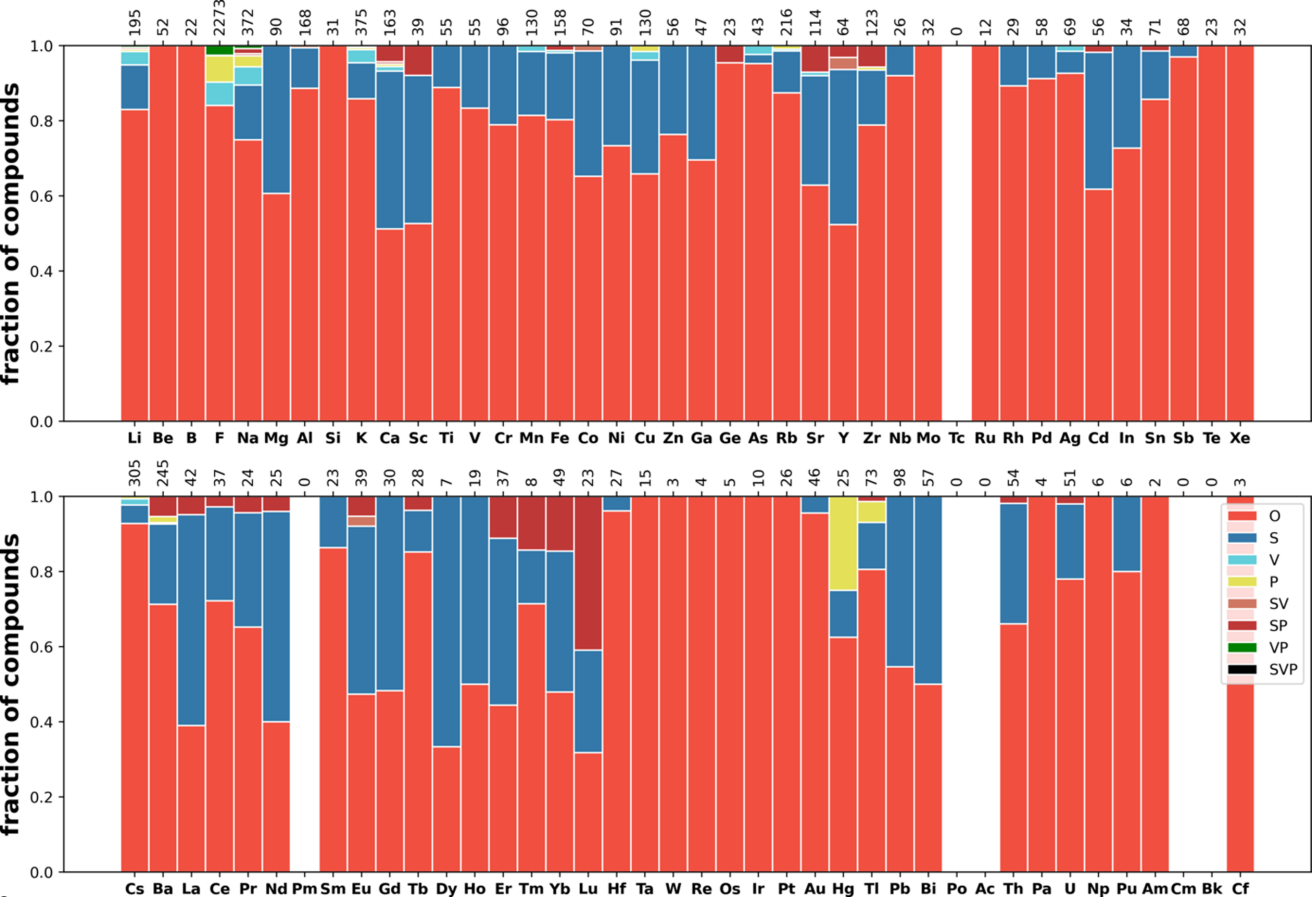
Distribution of disorder over elements in fluorides.

**Figure 12:**
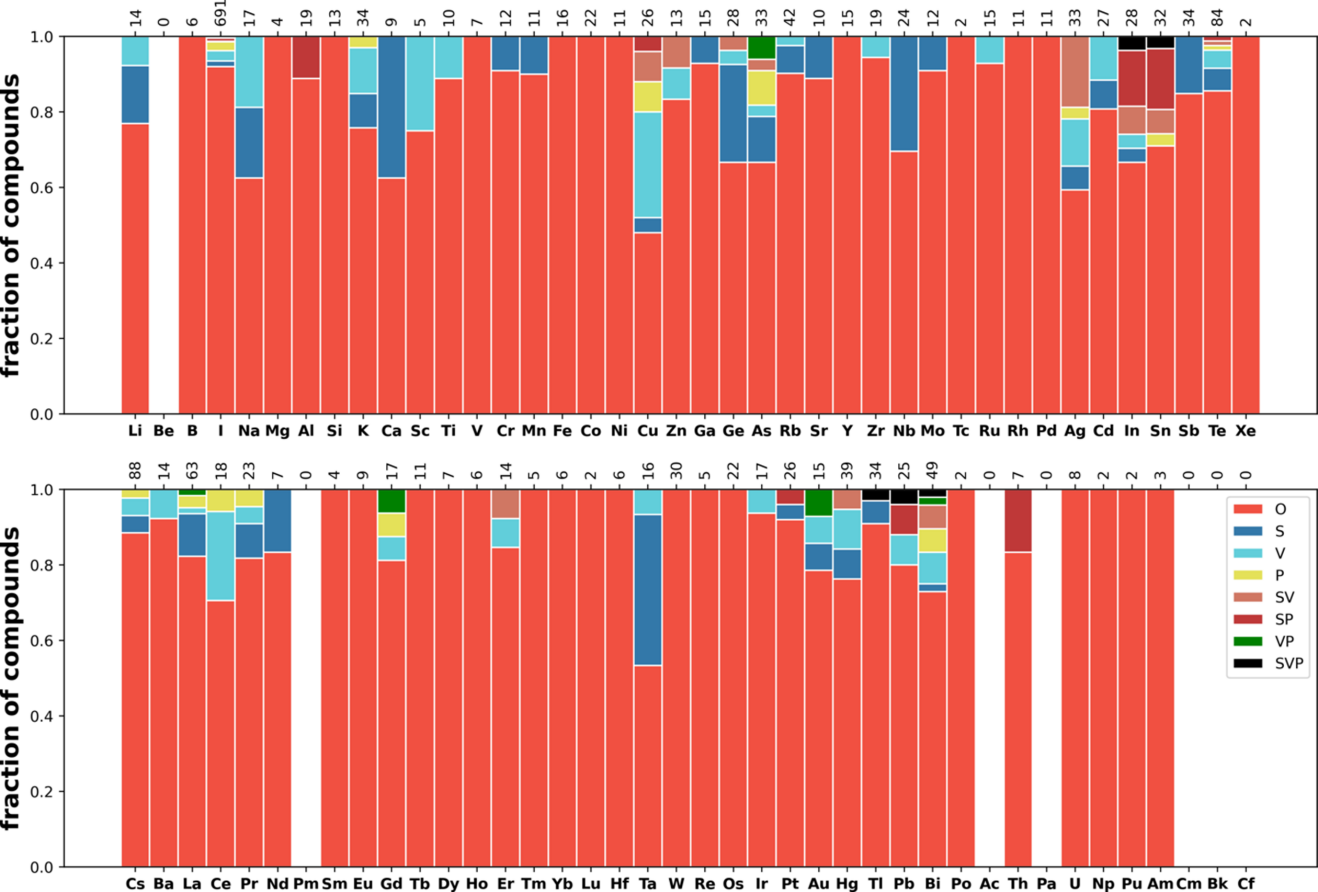
Distribution of disorder over elements in iodides.

**Table 1:** Rules to determine the disorder type of an orbit N/A corresponds to parameters that are not applicable to the definition of that orbit type.

Line No.	No. of elements	Total occupancy	Internal intersection	External intersection	Total occupancy of the combined site	Same intersecting element	Type of orbit
1	1	1	N/A	N/A	N/A	N/A	O
2	1	< 1	False	False	N/A	N/A	V
3	> 1	≥ 0.989	False	False	N/A	N/A	S
4	> 1	< 0.989	False	False	N/A	N/A	SV
5	1	< 1	True	False	≥ 0.989	N/A	P
6	1	< 1	True	False	< 0.989	N/A	VP
7	> 1	< 1	True	False	≥ 0.989	N/A	SP
8	> 1	< 1	True	False	< 0.989	N/A	SVP
9	1	< 1	N/A	True	≥ 0.989	True	P
10	1	< 1	N/A	True	< 0.989	True	VP
11	≥ 1	< 1	N/A	True	≥ 0.989	False	SP
12	≥ 1	< 1	N/A	True	< 0.989	False	SVP

**Table 2:** Examples of orbit classification The figures referred to are all in the supporting information.

No.	Formula	Collection code	Orbit types	Figure
1	Fe_3_Mn_4_Ge_6_	74	{S, O}	S1
2	Sm_0.3_Ce_0.7_O_1.85_	28793	{S, V}	S2
3	Sr_2_NiN_2_	91272	{P, P, O}	S3
4	(Ca_0.4_Eu_0.1_Gd_0.3_)WO_4_	253699	{O, O, SV}	S4
5	Ca_2_Co_12_As_7_	94411	{O, V, P}	S5
6	Ca_0.82_F_2.36_Th_0.18_	202039	{VP, S}	S6
7	Ga_2_Te_3_	67709	{V}	S7
8	RbCr(SO_4_)_2_	173671	{O, P}	S8
9	Ca_0.97_Co_0.199_Mg_0.831_Si_2_O_6_	74470	{O, S, SP}	S9
10	Al_12_Br_0.54_Cs_6.559_Na_4.43_O_48_Si_12_Zr_0.091_	6319	{O, V, S, SVP}	S10
11	Li_7_Zn_0.48_SiS_6_	116351	{O, V, SP}	S11
