# Supplementary material for: Classification and statistical analysis of structural disorder in crystalline materials
Source: J Appl Crystallogr. 2025 May 29;58(Pt 3):659–77. doi: 10.1107/S1600576725003000 (PMC12135987; doi:10.1107/S1600576725003000)
Supplement: Supplementary file 1 [file j-58-00659-sup1.pdf]

JOURNAL OF APPLIED CRYSTALLOGRAPHY

Volume 58 (2025)

Supporting information for article:

Classification and statistical analysis of structural disorder in crystalline materials

Dmytro Antypov, Chris M. Collins, Matthew S. Dyer, John B. Claridge and Matthew J. Rosseinsky

Supporting Information:
Statistical analysis of structural disorder in crystalline materials

Dmytro Antypov, Chris M. Collins, Matthew S. Dyer,
John B. Claridge, Matthew J. Rosseinsky
email: m.j.rosseinsky@liverpool.ac.uk

Contents

1 Glossary **1**

2 Examples of orbit classification **3**

3 Choice of the site intersection criteria **8**

4 Distribution of disorder over elements for SV, SP, VP, and SVP disorder. **12**

5 Distribution of disorder over elements for different types of compounds. **14**

1 Glossary

- **CIF** - crystallographic information file
- **ICSD** - Inorganic Crystal Structure Database
- **crystallographic orbit** - a set of points which are generated from one site by the symmetry operations of the space group G
- **O** - ordered crystallographic orbit
- **S** - substitutionally disordered orbit
- **V** - orbit with vacancies
- **P** - positionally disordered orbit
- **SV** - subtitutionally disordered orbit with vacancies
- **SP** - substitutionally and positionally disordered orbit
- **VP** - positionally disordered orbit with vacancies
- **SVP** - orbit which has substitutional, positional, and vacancy disorder
- **intersecting sites** - two sites intersect if the distance between them $r_{ij} < max\{1, 0.5\,(R_i + R_j)\}$, where the distance is measured in angstroms, R_i and R_j are average ionic radii of species occupying the i-th and j-th sites (or atomic radii, if ionic radii are not available)

– **internal intersection** - at least two sites which belong to the same orbit intersect

– **external intersection** - at least two sites which belong to different orbits intersect

– **combined orbit** - a combined orbit is a union of externally intersecting orbits

– **combined site** - a combined site is a site of a combined orbit, it is composed of intersecting sites which can be isolated from other sites. It also refers to the site composed of intersecting sites of the orbit with internal intersection

– **combined site multiplicity** - the multiplicity of a combined site is a number of combined sites in an orbit with intersections

– **total occupancy of the i-th element for combined site** - the sum of all occupancies for the i-th element within the combined site. This value is used for calculation of mixing entropy for positionally disordered orbits (P, SP, VP, SVP)

– **total occupancy of the combined site** - the sum of all occupancies for all elements within the combined site. If this value is not equal to one within the small tolerance factor (taken as 0.011 in this paper) then it indicates the presence of vacancies

– **substitutional disorder** - disorder due to more than one chemical element occupying a crystallographic or combined site. Noted by adding "S" to the orbit label

– **positional disorder** - disorder due to more than one position available for atoms to occupy within a combined site. Noted by adding "P" to the orbit label

– **vacancies** - a crystallographic or combined site contains vacancies if its total occupancy is not reaching 1. Noted by adding "S" to the orbit label

– **mixing entropy** - the entropy due to the presence of different crystal configurations due to substitutional disorder or vacancies (positional disorder does not contribute to mixing entropy)

– **configurational entropy** - the entropy due to the presence of different crystal configurations due to substitutional disorder, vacancies, and/or positional disorder

– **fraction of disordered sites with X disorder** - the fraction of disordered sites with X disorder type (were X can be any type of disorder, S, V, P, SV, SP, VP, SVP)

– **compound disorder set** - the set of all orbit disorder types characterising a compound

– **compound disorder class** - compounds can be aggregated into either non-intersecting classes O ({O} disorder set), S ({S} and {O,S} disorder sets), V({V} and {O,V} disorder sets), P({P} and {O,P} disorder sets), M (all other disorder sets), or intersecting classes O (compound has at least one O orbit), S (compound has at least one of S, SV, SP, or SVP orbit), V (compound has at least one V, SV, VP, or SVP orbit), P (compound has at least one P, VP, SP, or SVP orbit)

– **Pettifor scale** - a one-dimensional ordering of elements based on their ability to substitute each other in ordered compounds without changing the structure type

– **a duplicate** - we consider two compounds to be duplicates if they have the same chemical formula and the same space group number

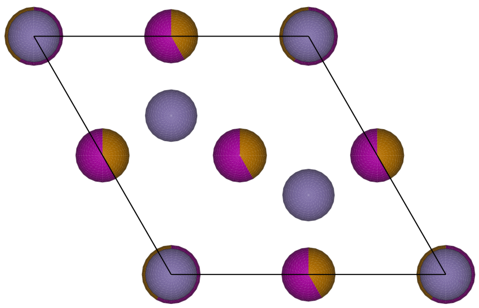

Figure S1: Example of a compound with substitutional disorder (S). Iron manganese germanide (3/4/6), $Fe_3Mn_4Ge_6$, Collection code 74 [1]. Colour code: ochre Fe, magenta Mn, violet Ge.

– **oxide** - we consider oxide as a compound containing oxygen which do not contain hydrogen and any other anion except oxygen. Similar definitions are used for carbides, nitrides, fluorides, phosphates, sulphides, chlorides, selenides, bromides, and iodides

– **intermetallics/alloys** - we consider compounds containing only metals in their composition formula

2 Examples of orbit classification

Figure S1 shows iron manganese germanide (3/4/6), $Fe_3Mn_4Ge_6$, Collection code 74 [1], iron manganese germanide (3/4/6) has 5 orbits. Three of them are ordered (O) and occupied by Ge. The other two are substitutionally disordered (S) and are shared by Mn and Fe: on both orbits, the occupancy of Fe is 0.4286 and the occupancy of Mn is 0.5714.

Figure S2 shows samarium cerium(IV) oxide, $Sm_{0.3}Ce_{0.7}O_{1.85}$, Collection code 28793 [2]. Samarium cerium(IV) oxide has substitutional disorder (S) and vacancies (V). Sm and Ce are located on the same orbit with occupancies 0.3 and 0.7 respectively, so that the total occupancy is 0.3+0.7=1. This orbit does not have internal or external intersection, so it is classified as S. Oxygen is located on another orbit. Since this orbit is occupied only by oxygen, its total occupancy is $0.925 < 1$ and there is no internal or external intersection (no positional disorder), this orbit is classified as V.

Figure S3 shows examples of ordered orbits and orbits with positional disorder. In distrontium nickel nitride, Sr_2NiN_2, collection code 91272 [3], Sr^{2+} ions are located on the ordered orbit (O) which is characterised by one element occupying it with occupancy 1. Ni^{2+} are located on a positionally disordered orbit (P), because there is internal intersection of sites of this orbit each occupied with probability 0.5 and the occupancy of the combined site is $2 \cdot 0.5 = 1$, thus there are no vacancies. An analogous classification can be applied to the orbit containing N^{3-} species, which is also positionally disordered (P).

Figure S4 shows an example of SV orbit that corresponds to the mixture of substitutional disorder and vacancies. Calcium europium gadolinium tungstate, $(Ca_{0.4}Eu_{0.1}Gd_{0.3})WO_4$, collection code 253699 [4], has two ordered orbits occupied by respectively O and W. Ca, Eu, Gd are located on another orbit with occupancies 0.4, 0.1, 0.3 respectively, so that the total occupancy is $0.4 + 0.1 + 0.3 = 0.8 < 1$. There is no internal or external orbit intersection. This orbit is classified as SV.

Figure S5 shows an example of a P orbit that is obtained by the external intersection of two crystallographic orbits. In calcium cobalt arsenide, $Ca_2Co_{12}As_7$, collection code 94411 [5], cobalt is located on three orbits, one of which is fully occupied and therefore ordered, and the other two have occupancies 0.46 and

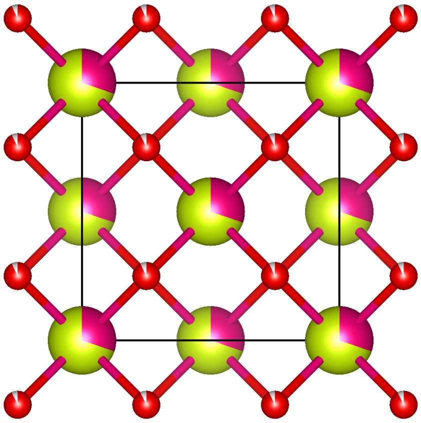

Figure S2: Example of a compound with substitutional disorder (S) and vacancies (V). Samarium cerium(IV) oxide, $Sm_{0.3}Ce_{0.7}O_{1.85}$, Collection code 28793 [2]. Colour code: pink Sm, yellow Ce, red O.

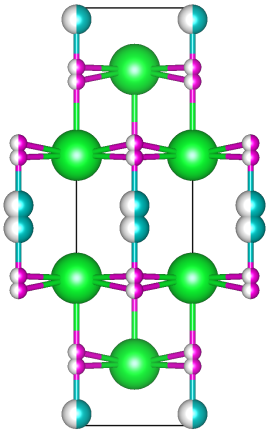

Figure S3: Example of a compound with positional disorder P. Distrontium nickel nitride, Sr_2NiN_2, Collection code 91272 [3]. Colour code: green Sr, cyan Ni, magenta N.

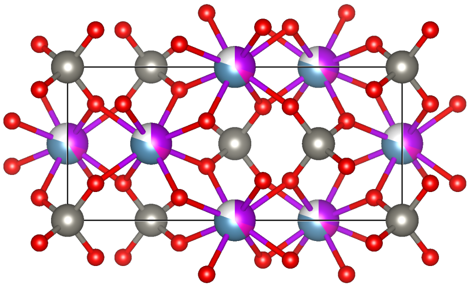

Figure S4: Example of a compound with positional disorder SV disorder. Calcium europium gadolinium tungstate, $(Ca_{0.4}Eu_{0.1}Gd_{0.3})WO_4$, collection code 253699 [4]. Colour code: blue Ca, magenta Eu, purple Gd, grey W, red O.

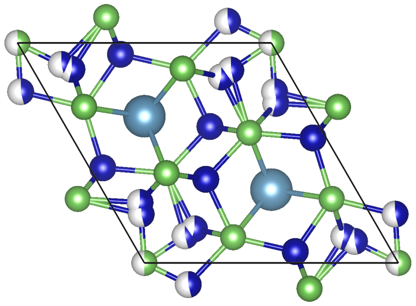

Figure S5: Example of a compound with P orbit formed by external intersection of two orbits. Calcium cobalt arsenide, $Ca_2Co_{12}As_7$, collection code 94411 [5]. Colour code: light blue Ca, dark blue Co, green As.

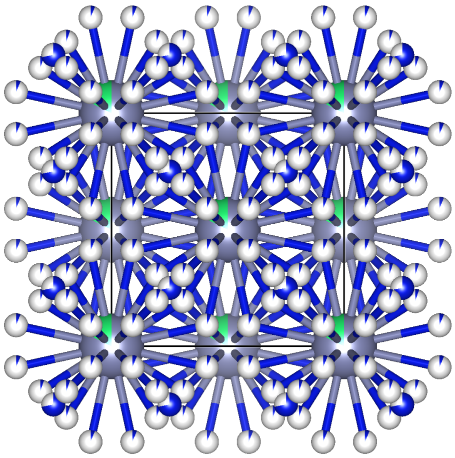

Figure S6: Example of a compound with VP orbit corresponding to location of fluoride in interstitial positions of ion conducting material. Chemical Formula $Ca_{0.82}F_{2.36}Th_{0.18}$, collection code 202039 [6]. Colour code: Ca grey, Th green, F blue.

0.54 respectively. Since these partially occupied orbits intersect and form one positionally disordered orbit with the occupancy of the combined site of $0.46 + 0.54 = 1$, this orbit is classified as P.

An interesting example of an unusual orbit classified by our code as VP is shown in Figure S6. In $Ca_{0.82}F_{2.36}Th_{0.18}$, collection code 202039 [6] fluoride occupies three overlapping orbits inside interstitial voids. All sites of these orbits intersect forming a continuous network of intersecting sites so that there is only one connected component. This connected component in our terms represents one very delocalised combined site with occupancy 9.34 (as there are 4 formula units in a unit cell). Our framework classifies this case as a VP rather than a simply P orbit, because the occupancy of the combined site is not 1 within the tolerance threshold and all three orbits are occupied by a single element F. Potentially we could separate such cases in a distinct class, but we do not do it here, leaving it for the future. It is an interesting example as all compounds with 'liquid-like' sublattices, such as archetypal AgI fall into this category.

Figure S7 shows an example of a compound with vacancies. In gallium telluride, Ga_2Te_3, collection code 67709 [7], both Ga and Te partially occupy their orbits with occupancies respectively 0.5 and 0.75, however there are no internal or external intersections, so both orbits are classified as V.

Figure S8 shows Rubidium chromium bis(sulfate), $RbCr(SO_4)_2$, collection code 173671 [8], which represents a common example of positional disorder of oxygen due to the external intersection of crystallographic orbits occupied solely by oxygen. In this case most of oxygen atoms are located on one of the two orbits with

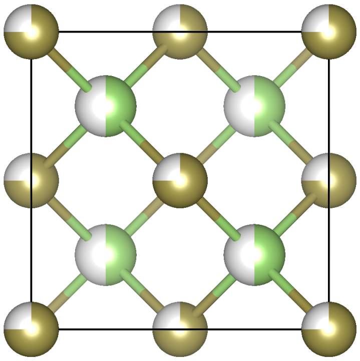

Figure S7: Example of a compound with vacancies. Gallium telluride, Ga_2Te_3, Collection code 67709 [7]. Colour code: Ga green, Te gold.

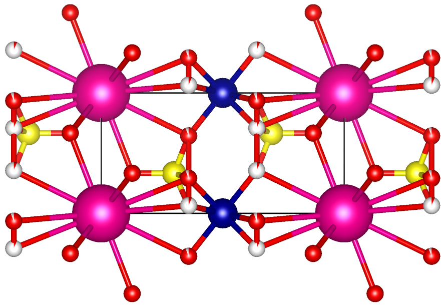

Figure S8: Common example of positional disorder due to external intersection of oxygen orbits. Rubidium chromium bis(sulfate), $RbCr(SO_4)_2$, collection code 173671 [8]. Colour code: Rb magenta, Cr blue, S yellow, O red.

occupancy 0.936 while the occupancy of the other orbit is 0.064. These two orbits intersect with each other forming pairs of combined sites sites with a total occupancy of one. Therefore, these orbits are classified as positional disorder P.

Figure S9 shows an example of an SP orbit formed by external intersection of two orbits occupied by different elements. In $Ca_{0.97}Co_{0.199}Mg_{0.831}Si_2O_6$, Collection code 74470 [9], Ca and Co are located on two very close orbits with occupancies 0.97 and 0.03 respectively. Since these two orbits externally intersect with the occupancy of the combined site $0.97 + 0.03 = 1.0$, this orbit is classified as SP.

Figure S10 shows an example of an SVP orbit. In $Al_{12}Br_{0.54}Cs_{6.559}Na_{4.43}O_{48}Si_{12}Zr_{0.091}$, collection code 6319 [10], three orbits externally intersect. Two of them are occupied by Cs and the other one by Na. Since the occupancy of the combined site $0.257 + 0.09 + 0.554 = 0.9$ is less than one, this orbit is classified as SVP.

Figure S11 shows an example of a complex Li argyrodite compound [11], $Li_7Zn_{0.48}SiS_6$, which has S, V, and SV types of disorder. In this compound, among the total of 40 orbits, there are 3 ordered orbits containing Si, 15 ordered orbits containing S and 22 orbits containing Li with various occupancies: 9 ordered (O), 7 orbits contain vacancies (V), 2 substitutionally disordered orbits (S) containing Li and Zn and 4 orbits of SV type containing Li, Zn and vacancies. There are no internal or external intersections.

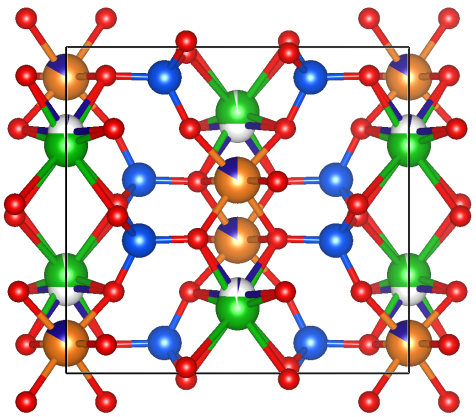

Figure S9: Example of the SP orbit formed by external intersection of two orbits occupied by different elements. $Ca_{0.97}Co_{0.199}Mg_{0.831}Si_2O_6$, collection code 74470 [9]. Colour code: Ca green, Co dark blue, Si blue, Mg orange, O red.

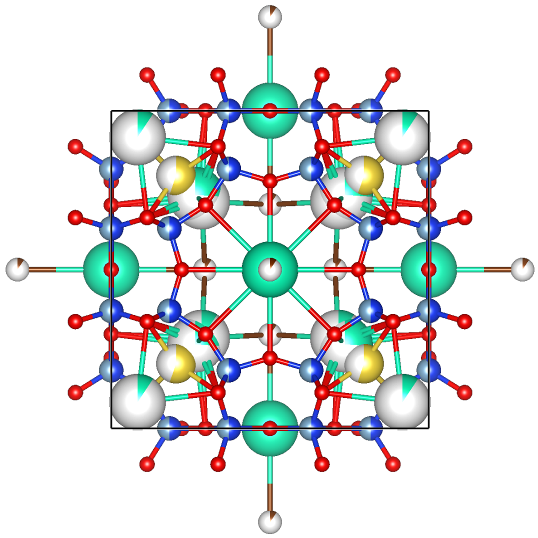

Figure S10: Example of an SVP orbit formed by external intersection of three orbits occupied by different elements and non integer intersection number. $Al_{12}Br_{0.54}Cs_{6.559}Na_{4.43}O_{48}Si_{12}Zr_{0.091}$, collection code 6319 [10]. Colour code: Si blue, Al light blue, O red, Zr green, Cs turquoise, Na yellow, Br brown.

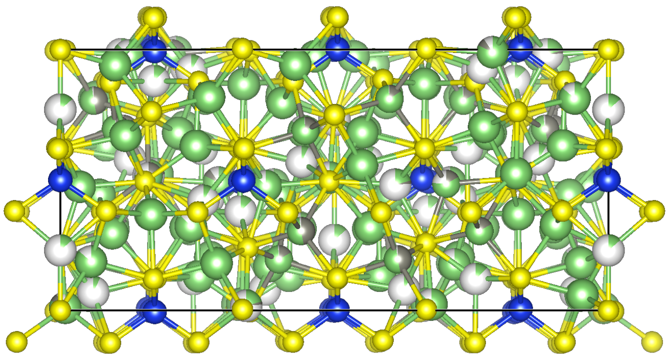

Figure S11: Example of a Li argyrodite compound, $Li_7Zn_{0.48}SiS_6$, which contains O, S, SV, V, and SVP orbits. Colour code: Li green, S yellow, Si blue, Zn grey.

3 Choice of the site intersection criteria

Some crystallographic structures contain intersecting atomic sites. If sites intersect they can not be occupied simultaneously, that is why we require that the sum of occupancies of intersecting sites is smaller than 1. We call this type of disorder positional disorder.

To detect a site intersection we consider two complementary approaches: (1) based on hard cut-off (2) based on sum of radii of species occupying the sites in question. In general, it is not obvious which method should work best because the interatomic distances depend strongly on the type of the atoms, type of bonding, the local coordination, distortions of the local coordination, occupancies, possible electronic effects (e.g., Jahn-Teller distortion, stereoactive lone pairs), spin state (in case of the first row transition metals) [12].

The hard cut-off idea is based on the observation that there is a lower limit of the size of species (atoms and ions) and the length of bonds. The shortest bonds are covalent between p-block elements, and p-block elements with hydrogen. If we exclude hydrogen, most of the remaining covalent bonds (C-C, C=C, C-O, N-O, etc.) have the length slightly above $1\mathring{A}$. So, taking the cut-off as $1\mathring{A}$, i.e. saying that all sites that are located closer than $1\mathring{A}$ intersect, seems to be a reasonable approach. To verify this statement, we plot the histogram of distances in compounds that have all sites fully occupied and those which have partial occupancies (Figure S12). One can see that the ordered compounds do not have distances smaller than about 1.1 $\mathring{A}$, meaning that indeed all sites located closer than 1.1 $\mathring{A}$ are positionally disordered sites.

The disadvantage of the $1\mathring{A}$ cut-off it that it may not detect intersections between sites occupied by larger species, i.e. metals. For example, Figure S13 shows the histogram of distances in ordered and disordered intermetallics. It can be seen, following the argument above, that the more reasonable cut-off for intermetallics would be within 1.75-1.9$\mathring{A}$ range. So, it would be good to make the cut-off sensitive to the type of species. This is explored below.

Another approach to define the intersection of two sites is to compare the distance between the sites with the sum of radii of species occupying the sites. The difficulty of this approach is that atomic/ionic radii are not well defined and depend on a large number of factors. In the literature different kinds of radii are available (atomic empirical, atomic calculated, ionic, covalent, Van der Waals), reflecting the strong dependence of the radii on the type of bonding. Traditionally the rule of a thumb for crystalline materials is to use ionic radii[12, 13] for elements with non-zero oxidation state and atomic radii for elements with zero oxidation states. However, as Shannon states in his paper ionic radii may deviate from tabulated values due to the reasons outlined above. Additionally, Shannon radii were determined from the distances in oxides and fluorides, and the radii in other ionic materials with smaller difference in electronegativities between anions and cations are expected to be more covalent in character (M-S, M-Cl, etc. bonds), which would make them shorter than the values expected from radii developed from oxides and fluorides. Shannon suggested that in some of these cases the lengths of bonds can be estimated by using a scaling factor proportional to the difference in electronegativities between anion and cation (see Figure S14). However, we are not aware of the sources of scaling factors for most of the element pairs. In some of those cases bond valence parameters are known, so distances, and correspondingly scaling factors, can be estimated using this approach [14]. Here we adopt a simpler approach in which we try to find a scaling factor for bond lengths which would leave all ordered compounds ordered. Then only the sites with distances between them which are smaller than this value would be marked as intersecting.

Figure S15 shows the dependence of the fraction of ordered materials containing spurious site intersections on the value of the scaling parameter α. There are two plots depending on which ionic radii is used for the ionic species. In one case, we choose the smallest ionic radius among all coordination numbers and spin states. In the other case, we take the largest radius among all coordination numbers and spin states. For

species with zero oxidation states, or those oxidation states which are not in Shannon tables, the empirical atomic radius is used. One can see that if the scaling factor is 1 (no scaling), more than 70% of structures contain intersection of sites. At $alpha = 0.5$, the intersections in a subset of ordered compounds reaches 0 (in fact it is 0.0016 due to the presence of mistakes in some structures). Interestingly, the scaling factor $alpha = 0.5$ works both for maximum and minimum ionic radii choices. It is interesting to note that for the visualisation of crystal structures in Vesta software, a similar scaling approach is used for the atomic radii but with a scaling factor of 0.4.

Applying these cutoffs to the random subset of 10,000 materials produces: 567 materials with intersections of sites, if hard cut-off $1\mathring{A}$ is used, and 546 if the sum of radii with scaling factor $alpha = 0.5$ is used. There are 143 structures for which the positional disorder assignment is different for these two methods. Among those, the 83 are classified as having intersecting sites using hard cut-off method, but having no intersection using scaled sum of radii. Correspondingly, there are 60 compounds with the opposite classification. It would be reasonable to assume that all intersections detected by hard cut off are intersections, but those which are detected by the sum of radii, but not detected by hard cut-off are also intersections (the opposite is not true as bonds can't be shorter than 1
A). Concluding, there are 567+60=627 compounds with intersection of sites. It is suggested to adopt the detection scheme in which the cut-off is determined as 0.5 of the sum of radii, but can't be smaller than 1
A.

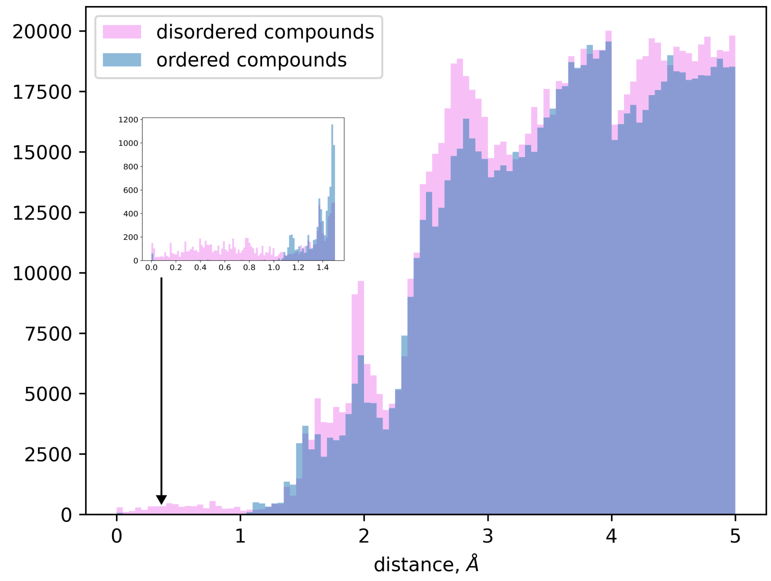

Figure S12: Histogram of inter atomic distances in 5000 ordered compounds (no partial occupancies) and 5000 compounds with partial occupancies.

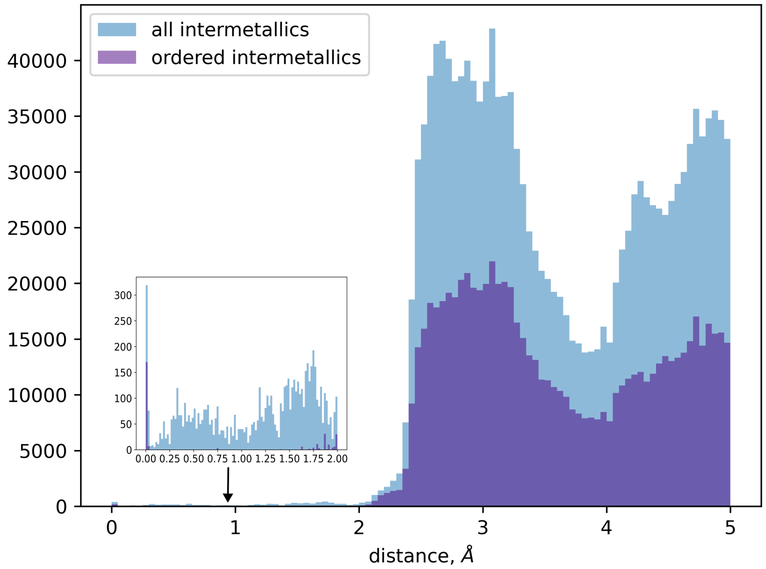

Figure S13: Histogram of inter atomic distances in completely ordered intermetallics (no partial occupancies) and intermetallics with partial occupancies.

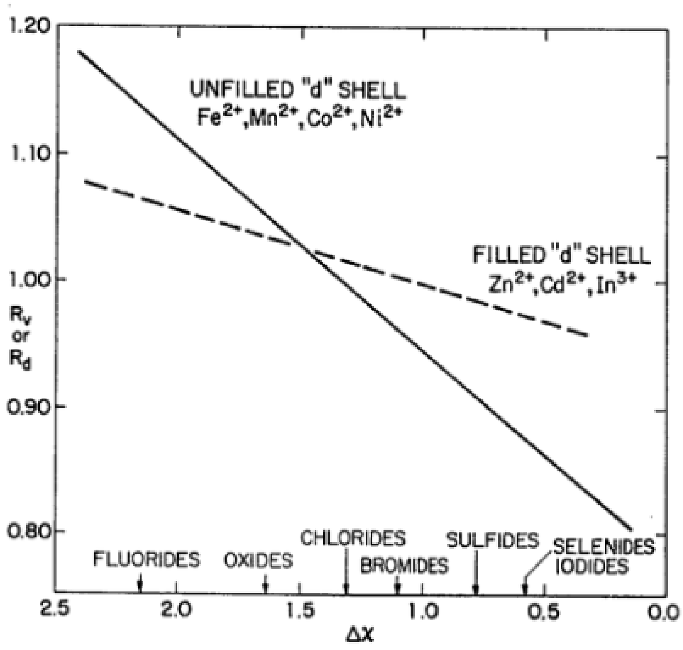

Fig. 7. Covalency contraction parameter, R_v or R_d, *vs* $\Delta\chi$ for filled and unfilled *d* shell cations.

Figure S14: Covalency contraction parameter for the first row transition metals. Reproduced from Shannon[12].

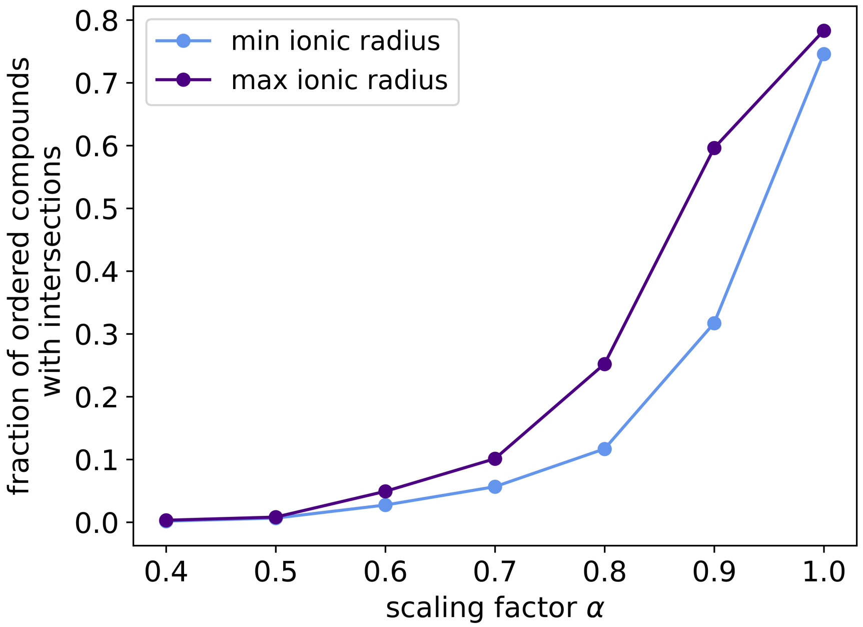

Figure S15: Dependence of the fraction of compounds with detected intersections on scaling factor α among 5000 random ordered compounds.

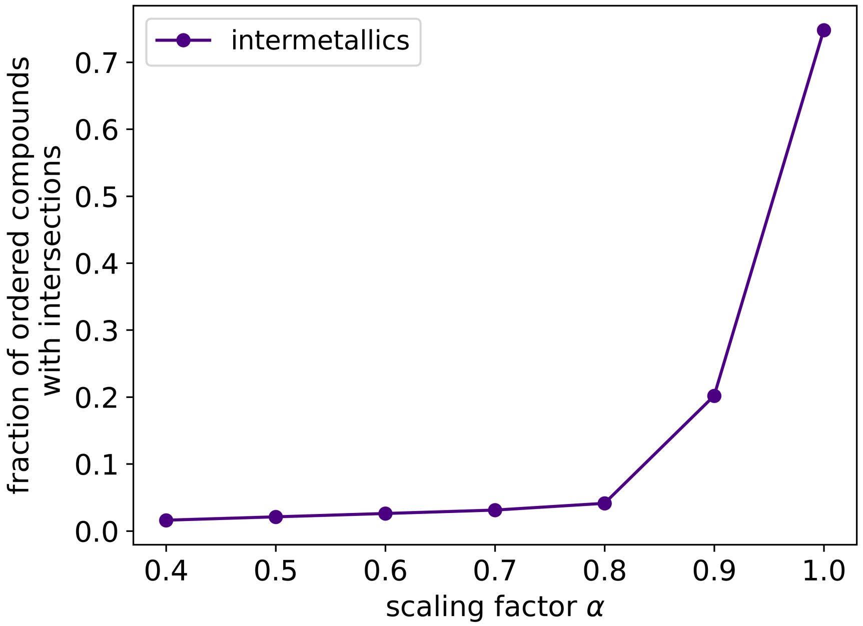

Figure S16: Dependence of the fraction of compounds with detected intersections on scaling factor α among 1000 random ordered intermetallics.

4 Distribution of disorder over elements for SV, SP, VP, and SVP disorder.

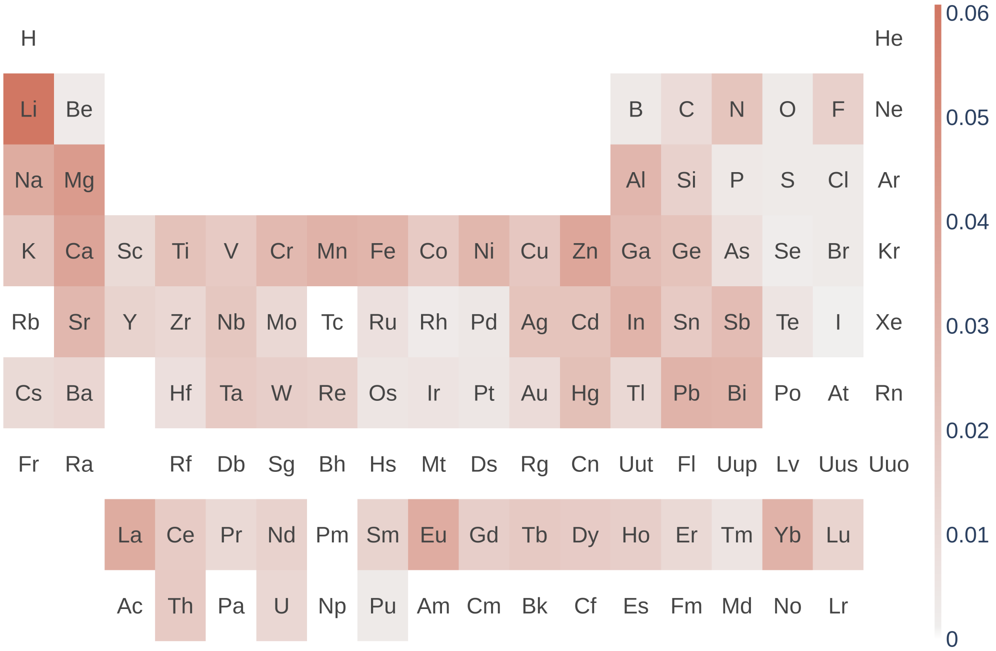

Figure S17: Distribution of SV type of disorder over the elements.

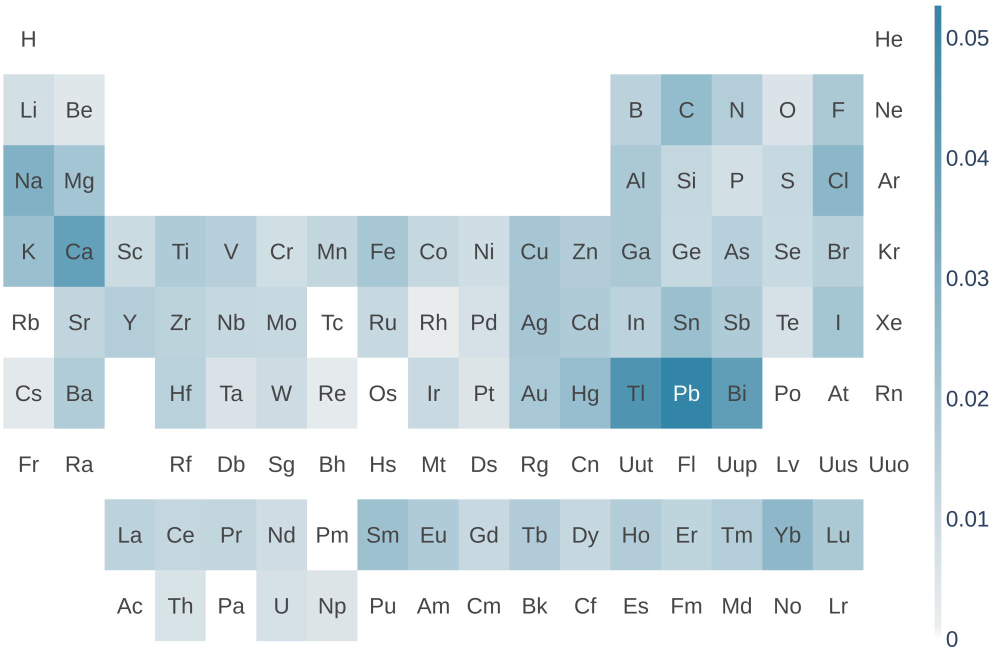

Figure S18: Distribution of SP type of disorder over the elements.

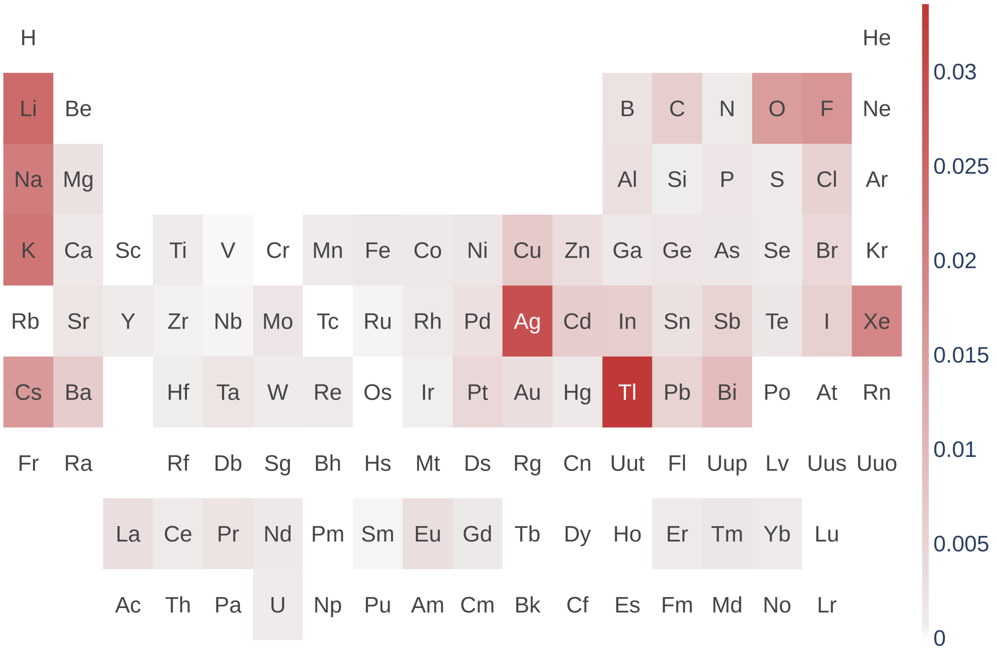

Figure S19: Distribution of VP type of disorder over the elements.

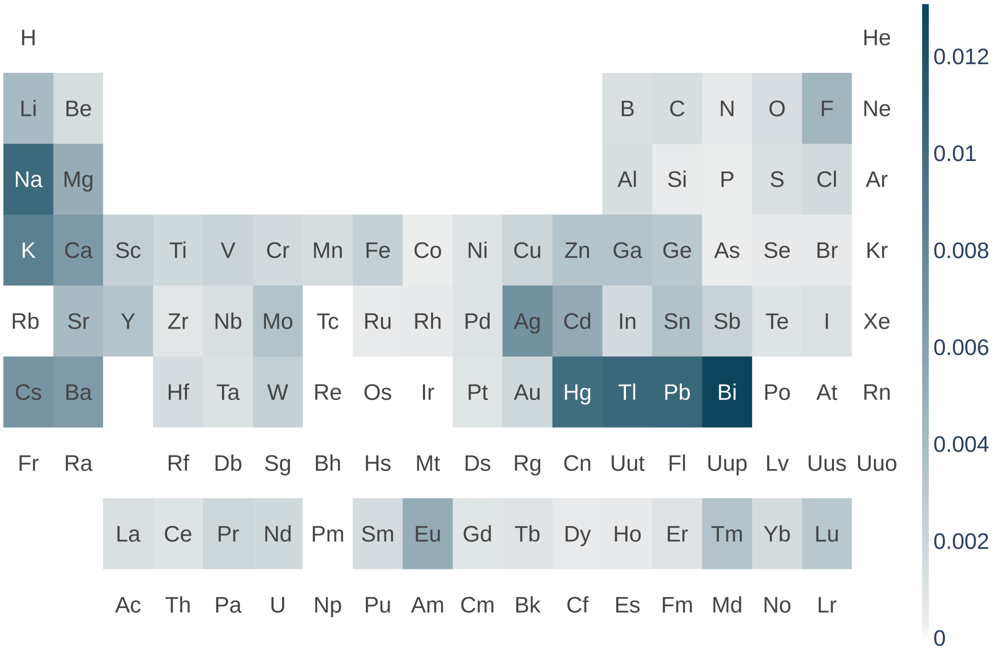

Figure S20: Distribution of SVP type of disorder over the elements.

5 Distribution of disorder over elements for different types of compounds.

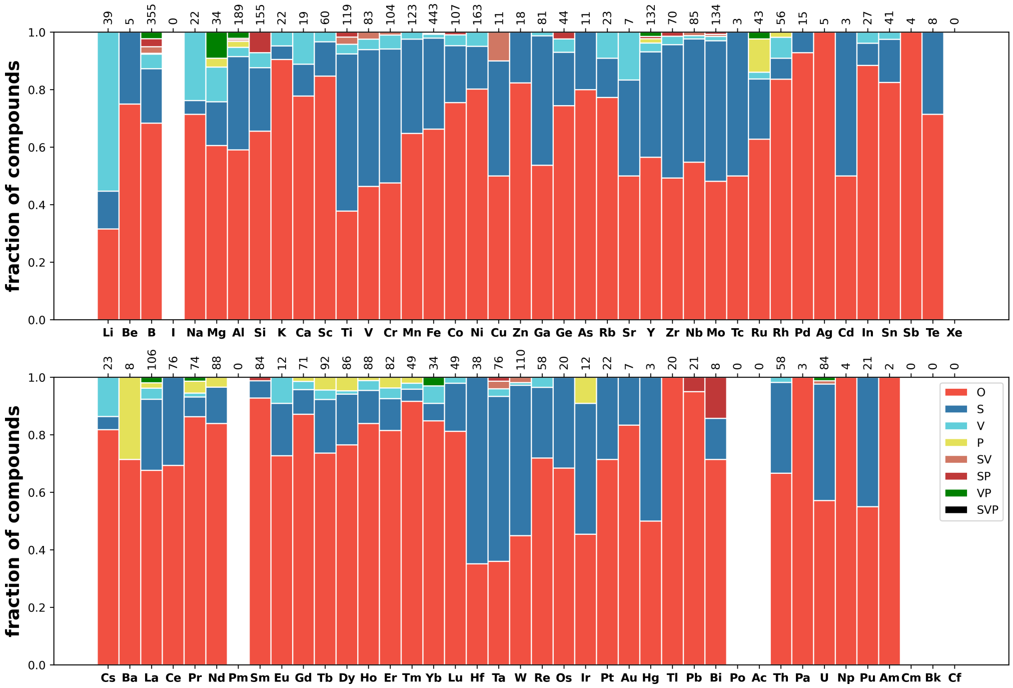

Figure S21: Distribution of disorder over the elements in carbides.

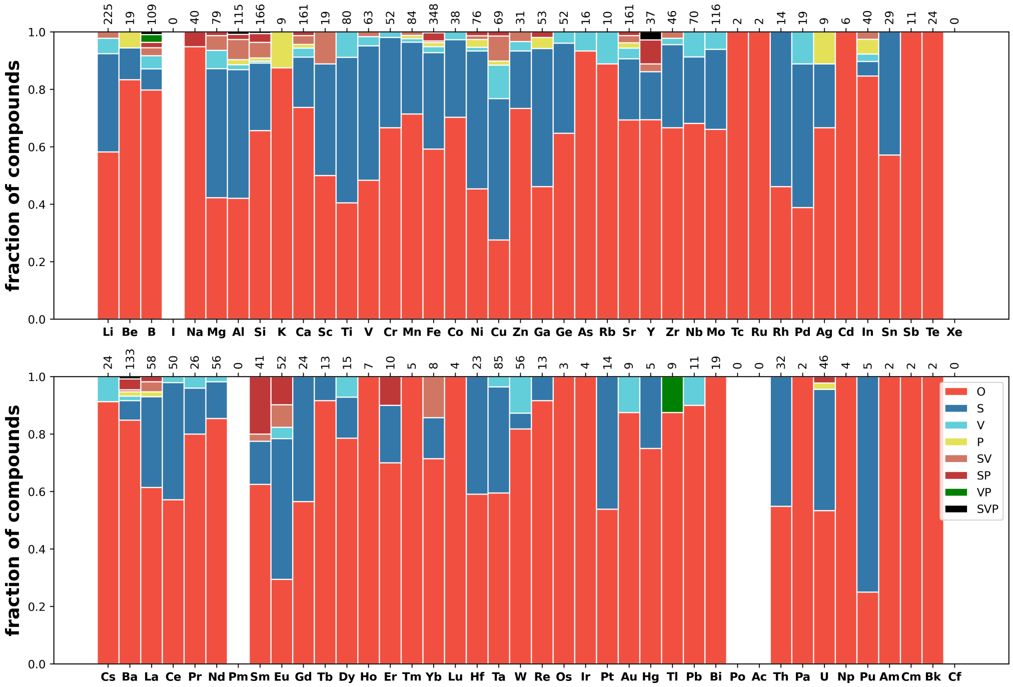

Figure S22: Distribution of disorder over the elements in nitrides.

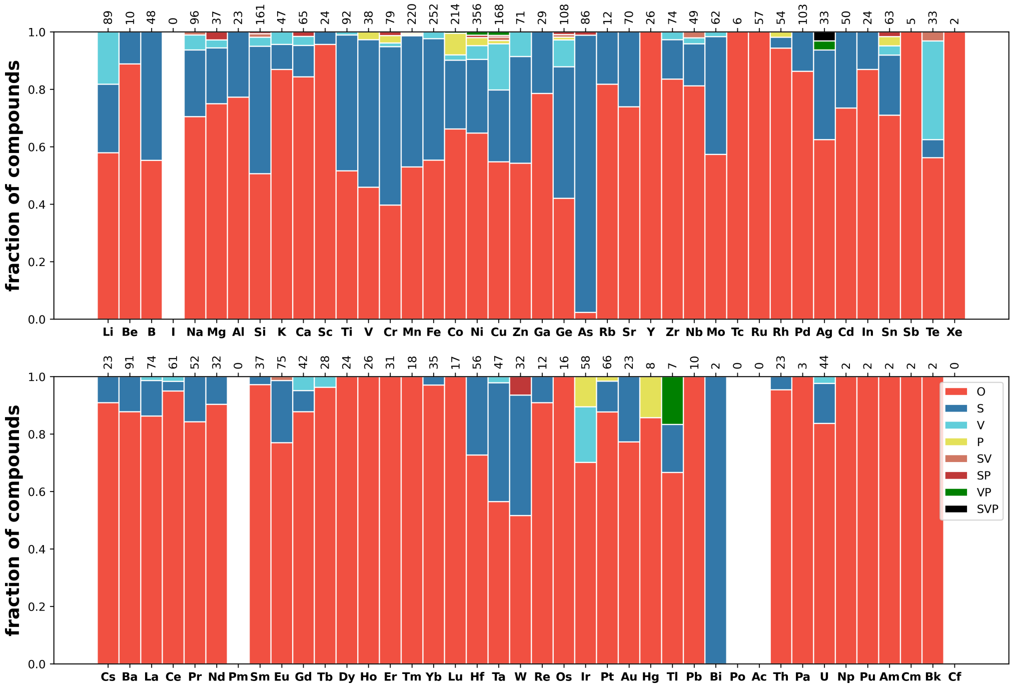

Figure S23: Distribution of disorder over the elements in phosphides.

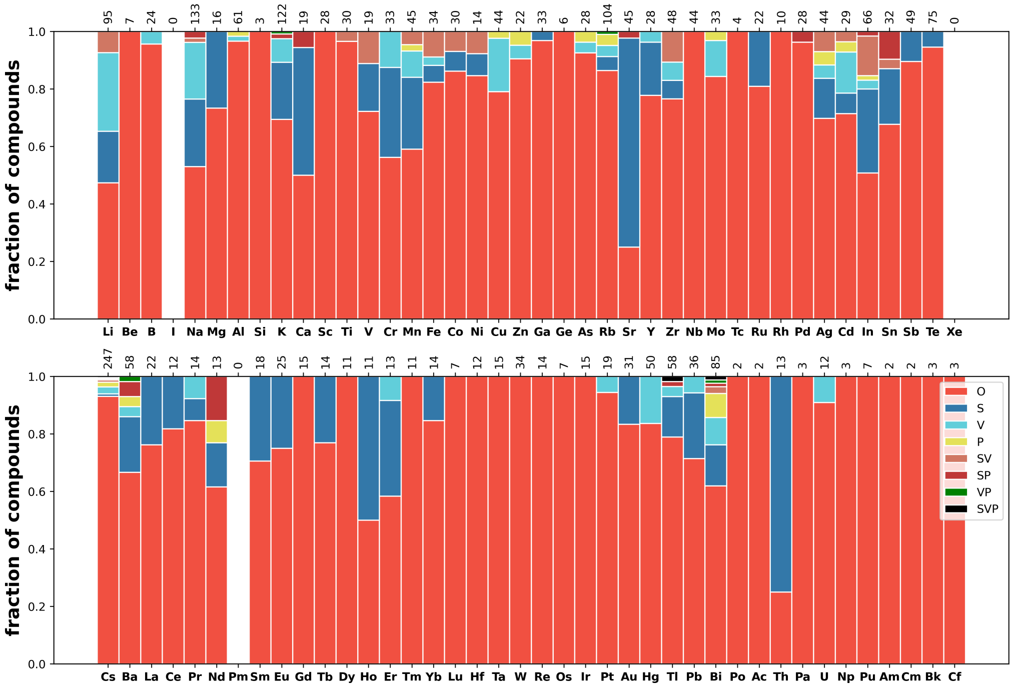

Figure S24: Distribution of disorder over the elements in chlorides.

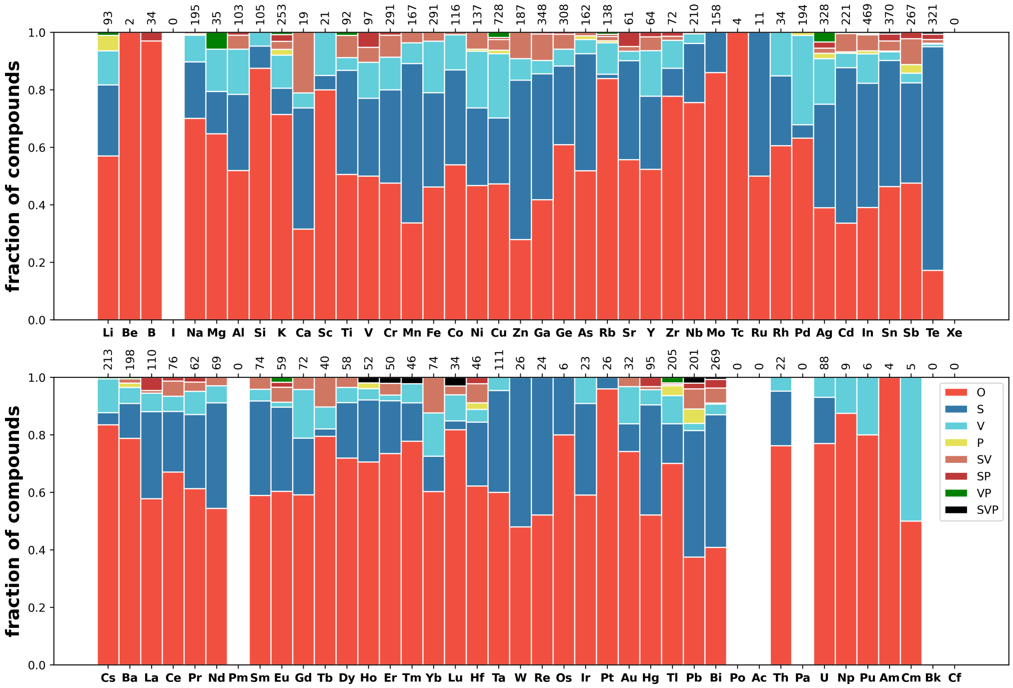

Figure S25: Distribution of disorder over the elements in selenides.

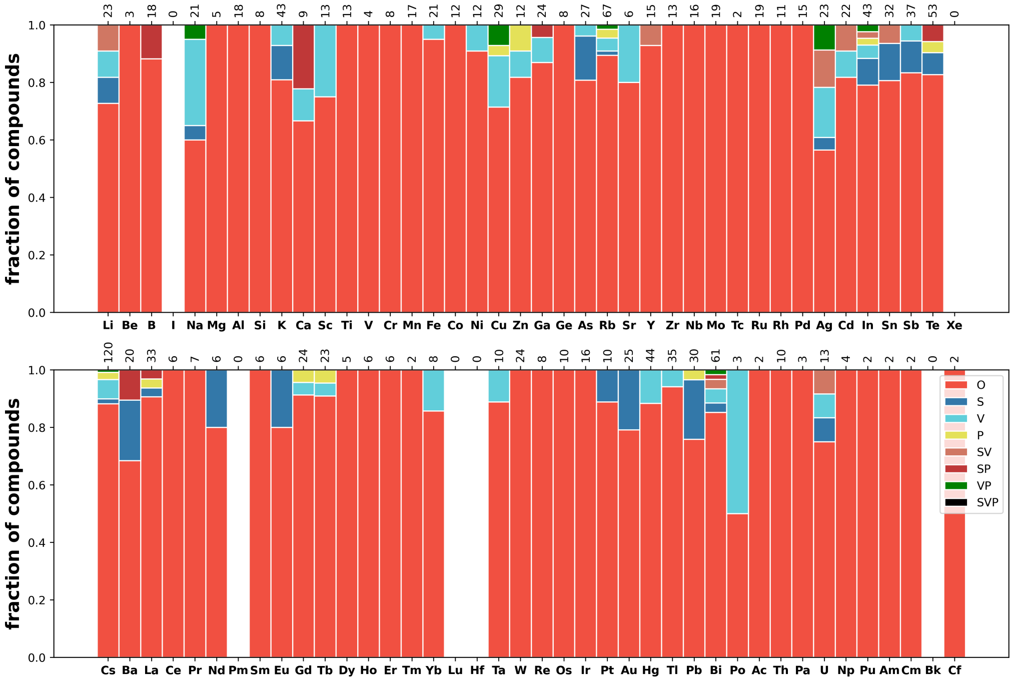

Figure S26: Distribution of disorder over the elements in bromides.

References

[1] B. Malaman, B. Roques, A. Courtois, and J. Protas. Structures cristallines de deux nouveaux germaniures ternaires: (Fe,Mn)7Ge6 et (Co,Mn)7Ge6. *Acta Crystallographica Section B Structural Crystallography and Crystal Chemistry*, 32(5):1352–1355, 5 1976.

[2] G Brauer and H Gradinger. Uber heterotype Nlischphasen bei Seltenerdoxyden. I. *Zeitschrift fur anorganische und allgemeine Chemie*, 276(5):209–226, 8 1954.

[3] Glen R. Kowach, N. E. Brese, U. M. Bolle, C. J. Warren, and F. J. Di Salvo. An unusual metallic nitride: Sr2NiN2. *Journal of Solid State Chemistry*, 154(2):542–550, 11 2000.

[4] Dmitry Batuk, Maria Batuk, Vladimir A. Morozov, Katrien W. Meert, Philippe F. Smet, Dirk Poelman, Artem M. Abakumov, and Joke Hadermann. Effect of cation vacancies on the crystal structure and luminescent properties of Ca0.851.5xGdxEu0.10.05+0.5xWO4 (0 x 0.567) scheelite-based red phosphors. *Journal of Alloys and Compounds*, 706:358–369, 2017.

[5] A. Hellmann and A. Mewis. Neue Erdalkalimetall-Phosphide und -Arsenide des Cobalts. *Zeitschrift fuer Anorganische und Allgemeine Chemie*, 627:1357–1364, 1950.

[6] J P Laval, A Mikou, B Frit, and J Pannetier. Neutron Diffraction Study of the Anion-Excess Fluorite-Related Ca(1-x)Th(x)F(2-2x) Solid Solution. *Journal of Solid State Chemistry*, 61:359–368, 1986.

[7] M. Guymont, A. Tomas, and M. Guittard. The structure of Ga2Te3 an x-ray and high-resolution electron microscopy study. *Philosophical Magazine A*, 66(1):133–139, 1992.

[8] D. V. West, Q. Huang, H. W. Zandbergen, T. M. McQueen, and R. J. Cava. Structural disorder, octahedral coordination and two-dimensional ferromagnetism in anhydrous alums. *Journal of Solid State Chemistry*, 181(10):2768–2775, 2008.

[9] Yasunori Tabira, Nobuo Ishizawa, and Fumiyuki Marumo. Cobalt atoms at M(2) site in C2/c clinopyroxenes of the system CaMgSi2O6 (Di)–CaCoSi2O6 (CaCoPx). *Mineralogical Journal*, 16(5):225–245, 1992.

[10] Joon Young Kim, Hyeon Seung Lim, Nam Ho Heo, Hong Joo Kim, and Karl Seff. Identification and structures of the X-ray induced luminescence centers in the zeolites Zr,X,Cs,Na-LTA, X = Cl, Br, and I. *Microporous and Mesoporous Materials*, 278:443–454, 4 2019.

[11] Bernhard T. Leube, Christopher M. Collins, Luke M. Daniels, Benjamin B. Duff, Yun Dang, Ruiyong Chen, Michael W. Gaultois, Troy D. Manning, Frédéric Blanc, Matthew S. Dyer, John B. Claridge, and Matthew J. Rosseinsky. Cation Disorder and Large Tetragonal Supercell Ordering in the Li-Rich Argyrodite Li7Zn0.5SiS6. *Chemistry of Materials*, 34(9):4073–4087, 4 2022.

[12] R D Shannon. Revised Effective Ionic Radii and Systematic Studies of Interatomie Distances in Halides and Chaleogenides. Technical report, 1976.

[13] R. D. Shannon and C. T. Prewitt. Effective ionic radii in oxides and fluorides. *Acta Crystallographica Section B Structural Crystallography and Crystal Chemistry*, 25(5):925–946, 5 1969.

[14] Ian David Brown. Recent developments in the methods and applications of the bond valence model. *Chemical Reviews*, 109(12):6858–6919, 12 2009.
